# Intraspecific variability in the filter mesh size of suspension feeding organisms: the case of invasive Ponto-Caspian corophiids (Crustacea: Amphipoda)

**DOI:** 10.7717/peerj.11245

**Published:** 2021-04-19

**Authors:** Péter Borza

**Affiliations:** Danube Research Institute, Centre for Ecological Research, Budapest, Hungary

**Keywords:** Character displacement, Ecological sexual dimorphism, Individual specialization, Niche differentiation, Ontogenetic niche shift

## Abstract

Suspension feeders play pivotal roles in the nutrient cycling of almost all aquatic ecosystems. Since sufficiently large differences in the filter mesh size (FMS) can lead to different food web positions, the inter- and intraspecific variability of this trait might be of community-level importance. The aim of this study was to quantify the range of FMS variation within the three invasive Ponto-Caspian *Chelicorophium* species based on a large material representing various conditions (1,224 specimens from 40 samples across Central Europe), characterize the components of variation within populations, identify the main factors determining intraspecific differences, and reveal how intraspecific variation affects the FMS overlaps among species. The FMS of the most widespread invader, *C. curvispinum*, varied within the broadest range (between 2.34–8.28 μm, compared to 2.51–5.97 μm in *C. robustum* and 1.08–3.23 μm in *C. sowinskyi*); nevertheless, the contribution of intraspecific plasticity to the invasion success of the species is not evident based on the present study. The within-individual variability of FMS increased with the individual mean of the trait and decreased with body size; however, it showed little differences among samples. The among-individual variation within samples could be partitioned into components related to body size (ontogenetic niche shift/differences among cohorts) and sex (ecological sexual dimorphism) as well as a seemingly random component (individual specialization), varying widely in extent and relative contributions. The FMS of *C. curvispinum* was significantly larger in the presence of *C. sowinskyi* than in allopatry, likely reflecting character displacement; however, it did not show further increase when *C. robustum* was also present. Similar differences could not be observed in *C. sowinskyi*. The FMS ranges of *C. curvispinum* and *C. robustum* never overlapped with that of *C. sowinskyi* in co-occurrence despite the considerable intraspecific differences among sites, suggesting that their interaction can be seen as a clear case of niche differentiation by food particle size. On the contrary, the strong overlaps observed between *C. curvispinum* and *C. robustum* indicate that other factors might play the primary role in their coexistence. The studied species appear to be suitable model organisms for identifying the drivers and mechanisms of FMS variability.

## Introduction

Suspension feeders play pivotal roles in the nutrient cycling of almost all aquatic ecosystems by virtue of their often high biomass and central position in food webs (Higgins & Vander Zanden, 2010; Atkinson et al., 2013, 2014). Therefore, revealing the dynamics of their trophic interactions is an important research objective especially in the context of local and global anthropogenic disturbances–such as climate change, invasive species, eutrophication, and microplastics–affecting the quantity, quality, as well as the size distribution of suspended matter substantially (Barnett, Adam & Lettenmaier, 2005; Higgins & Vander Zanden, 2010; Lewandowska et al., 2014; Abonyi et al., 2018, 2020; Germanov et al., 2018).

Comparing traits directly linked to resource acquisition offers a straightforward way for studying the trophic interactions and niche differentiation among functionally similar species. Nevertheless, even deeper insights might be gained by taking the intraspecific variation of the traits into account, as well (Bolnick et al., 2011; Violle et al., 2012; Des Roches et al., 2018). For example, the range (i.e., niche breadth) and components (i.e., within/among individuals, sexes, or size classes) of trait variation within species might influence the strength of interspecific interactions substantially (Bolnick et al., 2003, 2011). In addition, the spatial or temporal variability of the traits might be informative of the adaptive capacity and dynamics of the species (e.g., Jourdan et al., 2019; Santi et al., 2020).

Suspension feeders use several different methods for capturing food particles; nevertheless, most of these include regular meshes (i.e., filters; Riisgård & Larsen, 2010). In the simplest case, the filters are used for the mechanical retention of particles the size of which must be larger than the filter mesh size (henceforth ‘FMS’). Since sufficiently large differences in FMS among species can lead to qualitatively different diets and thus food web positions, the trait can be indicative of niche differentiation (Suh & Choi, 1998; Kang et al., 2009; Borza et al., 2018). Even if more complex mechanisms (e.g., adhesive forces) are involved, implying a less direct link with the size of the captured particles, FMS might still allow informative intra- or interspecific comparisons (Alstad, 1987).

Although the intraspecific variability of FMS might have community-level implications in several cases, especially when keystone species are involved, it has received relatively little attention so far. The most well-known in this regard are hydropsychid caddisflies which nonetheless represent a special case with respect to the adhesive nature of the silk material they use for constructing their nets and the possibility of repeated net spinning during the life of a single individual. Loudon & Alstad (1992) demonstrated experimentally that individual *Hydropsyche* sp. larvae decrease the mesh size and increase the total area of their nets as ambient current velocity increases (although this pattern was not observable in *Cheumatopsyche* sp. larvae). The plasticity of FMS has also been studied in *Daphnia* spp., where Lampert & Brendelberger (1996) found that individuals experimentally adapted to low food density have larger filter screens and finer meshes.

Corophiids are small (<10 mm) crustaceans, filtering suspended particles actively by pumping water through their self-constructed tubes attached to hard surfaces or burrowed into soft sediments. They are distributed worldwide in oceans and seas reaching high density especially in tidal mudflats (Gerdol & Hughes, 1994); however, some of the species also occur in freshwater, including three invasive *Chelicorophium* species (*C. curvispinum* (G.O. Sars, 1895), *C. robustum* (G.O. Sars, 1895), and *C. sowinskyi* (Martynov, 1924)) originating in the Ponto-Caspian region (Borza et al., 2015). Very few data have been published on their FMS and even less is known about the intraspecific variability of the trait. Borza et al. (2018) found that FMS showed body length and sex dependency within populations in the invasive *Chelicorophium* species within their native range (Lower Danube), and the proportion of unexplained within- and among-individual variation was different per species. Borza et al. (2018) found no evidence for site-related intraspecific differences; however, another study focusing on the oligohaline corophiids of the Baltic Sea revealed significant differences in the FMS of all three native species among sampling sites, and the FMS of the invasive Baltic population of *C. curvispinum* also differed considerably from that of the native population in the Lower Danube (Borza, Arbačiauskas & Zettler, 2021).

The aim of this study was to (1) quantify the range of FMS in the three invasive *Chelicorophium* species based on a large material representing a wide range of conditions, (2) characterize the components of variation within populations, (3) identify the main factors determining intraspecific differences, and (4) reveal how intraspecific variation affects the niche differentiation among species.

## Materials & methods

### The studied materials

**Figure 1:**
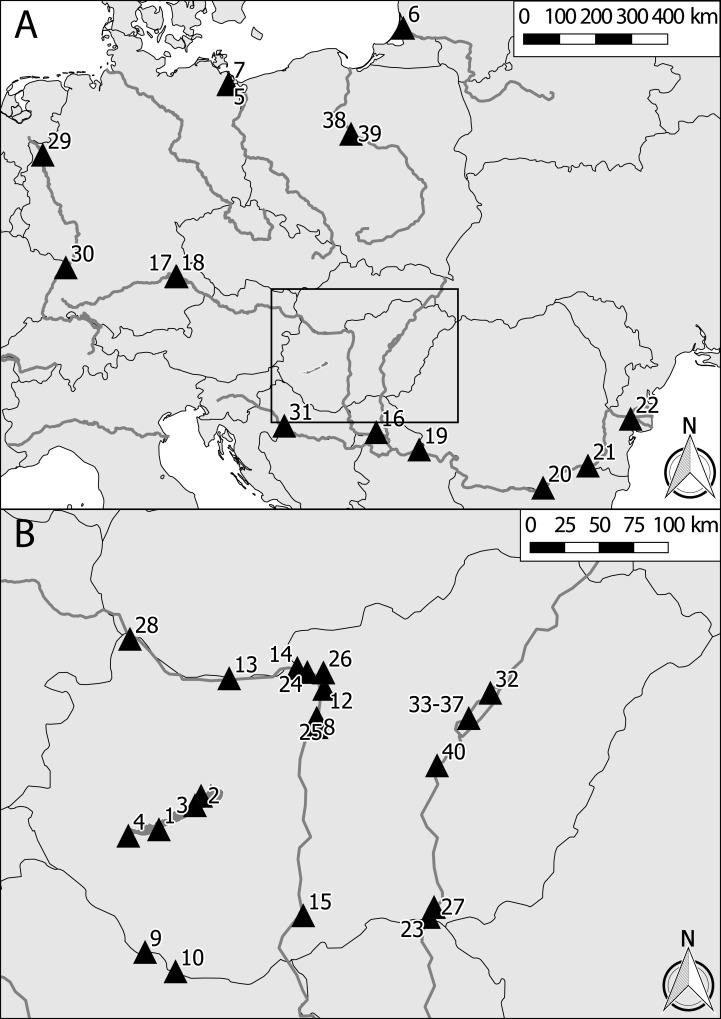
Map of the sampling sites. (A) Central Europe. (B) Hungary (marked by the rectangle in (A)). Site numbers as in Table 1.

Altogether 1,224 specimens (*N* = 715 for *C. curvispinum*, *N* = 202 for *C. robustum*, and *N* = 307 for *C. sowinskyi*) from 40 samples (stored in ethanol) across Central Europe were included in the analysis (Table 1, Fig. 1). The samples were chosen to represent various conditions regarding the locality and type of the waterbody, and season of year. Special emphasis was put on representing different species combinations.

**Table 1:** The samples included in the analysis.

Sample no.	Site	Water body	Date	Geographic coordinates	Species present	Studied materials					
						*C. curvispinum*		*C. robustum*		*C. sowinskyi*	
1	Fonyód	Lake Balaton	not available	46°45′07.8″N 17°33′16.4″E	C	24 (11/12/1)	1.8–5.2				
2	Paloznak	Lake Balaton	not available	46°58′32.7″N 17°56′59.5″E	C	23 (12/10/1)	2.2–4.7				
3	Tihany	Lake Balaton	not available	46°54′47.8″N 17°53′37.2″E	C	24 (14/9/1)	1.8–4.2				
4	River Zala mouth	Lake Balaton	not available	46°42′23.6″N 17°15′57.3″E	C	24 (14/10/0)	2.2–5				
5*	Peenestrom	Baltic Sea	20.06.1998	53°51′00.0″N 13°49′59.9″E	C	35 (19/16/0)	2.3–6.5				
6*	River Nemunas mouth	Baltic Sea	05.09.2015	55°20′12.1″N 21°14′53.4″E	C	30 (16/13/1)	2–5.5				
7*	Zecherin	Baltic Sea	25.09.2018	53°51′54.0″N 13°49′51.6″E	C	27 (13/10/4)	1.9–4.7				
8	Budapest	Middle Danube	20.04.2018	47°25′42.2″N 19°03′00.1″E	C-R-S	30 (15/15/0)	2.7–6.2	14 (8/6/0)	4-7.5		
9	Barcs	Middle Drava	09.11.2009	45°57′03.4″N 17°26′50.1″E	C-S	16 (8/8/0)	2.2–5			19 (16/3/0)	1.9–4.2
10	Vrbovka	Middle Drava	18.02.2017	45°49′41.5″N 17°44′06.5″E	C-S	24 (13/11/0)	3.2–5.1			21 (11/10/0)	3.1–4.7
11	Göd	Middle Danube	30.03.2007	47°40′40.3″N 19°07′29.2″E	C-S	24 (13/10/1)	2–6				
12	Göd	Middle Danube	12.10.2009	47°40′49.3″N 19°07′33.6″E	C-S	25 (13/12/0)	2.5–5.4				
13	Iza/Szőny	Middle Danube	24.08.2013	47°44′38.4″N 18°12′20.5″E	C-R-S	23 (17/6/0)	2.8–5.1	25 (16/8/1)	2-8.2		
14	Szob	Middle Danube	25.08.2013	47°48′53.6″N 18°51′50.6″E	C-R-S					24 (12/10/2)	1.9–5.2
15	Baja	Middle Danube	29.08.2013	46°12′04.1″N 18°55′30.7″E	C-R-S	24 (12/12/0)	2.2–5.1	24 (13/11/0)	3.3-6.6		
16	Novi Sad	Middle Danube	03.09.2013	45°15′41.8″N 19°53′13.7″E	C-R-S	24 (13/11/0)	2.1–5				
17	Geisling power plant (downstream)	Upper Danube	14.08.2013	48°58′26.0″N 12°21′44.0″E	C-R-S	24 (14/10/0)	2.3–5.6				
18	Geisling power plant (upstream)	Upper Danube	14.08.2013	48°58′44.9″N 12°19′56.9″E	C-R-S			24 (14/10/0)	3.1-7.6	24 (18/6/0)	2.5–5
19	Banatska Palanka/Bazias	Middle Danube	08.09.2013	44°48′18.3″N 21°23′23.7″E	C-R-S	24 (12/12/0)	2–4.8	24 (13/11/0)	3.1-6.7	24 (20/3/1)	1.7–4.6
20**	River Jantra mouth	Lower Danube	16.09.2013	43°40′26.9″N 25°37′10.0″E	C-R-S					20 (10/9/1)	1.8–4.5
21**	Chiciu/Silistra	Lower Danube	19.09.2013	44°07′07.3″N 27°14′04.4″E	C-R-S	24 (13/10/1)	2–5.3	27 (15/10/2)	2.1-7.7		
22**	Sf.Gheorghe arm	Lower Danube	25.09.2013	45°09′34.3″N 28°54′32.2″E	C-R-S	13 (6/7/0)	2.5–4.7	10 (7/3/0)	2.2-6.9	10 (5/5/0)	2.1–4.2
23	Tiszasziget	River Tisza	14.07.2019	46°11′07.9″N 20°06′16.8″E	C-R-S	24 (12/12/0)	2.3–5.5	24 (14/10/0)	2.6-8.2	24 (13/11/0)	2.3–5.6
24	Nagymaros	Middle Danube	17.07.1917	47°47′17.2″N 18°57′39.4″E	S					10 (6/4/0)	2.2–3.9
25	Budapest	Middle Danube	30.09.1932	47°29′21.7″N 19°03′05.7″E	S					10 (6/4/0)	2.3–5.5
26	Vác	Middle Danube	30.09.1930	47°47′05.6″N 19°07′00.9″E	S					5 (4/1/0)	3.1–4.3
27	Szeged	River Tisza	9-10.1943	46°15′00.3″N 20°09′16.6″E	S					25 (11/10/4)	1.3–4.1
28	Rajka	Middle Danube	28.05.2003	47°59′25.0″N 17°14′17.4″E	C-S	21 (13/7/1)	1.5–6.3			25 (21/3/1)	1.6–5.5
29	Ossenberg	River Rhein	28.05.2013	51°35′08.0″N 6°35′55.1″E	C-R-S	2 (1/1/0)	2.8–5.6	24 (11/11/2)	2-7.9		
30	Rastatt	River Rhein	8.2011	48°53′15.8″N 8°08′12.6″E	C-R-S	22 (13/9/0)	2.8–4.8	6 (4/2/0)	3.7-7.5		
31	Kratecko	River Sava	10.09.2016	45°23′57.6″N 16°37′22.0″E	C-S	24 (13/11/0)	2–4.7			24 (11/10/3)	1.4–4.9
32	Tiszafüred	River Tisza	03.08.2019	47°38′25.4″N 20°43′37.7″E	C-S	24 (12/12/0)	2.1–4.1			3 (2/1/0)	3.3–3.7
33	Kisköre	River Tisza	14.05.2013	47°28′42.1″N 20°30′49.5″E	C-S	19 (9/10/0)	4.3–6.2			20 (10/10/0)	3.7–5.3
34	Kisköre	River Tisza	31.07.2013	47°28′42.1″N 20°30′49.5″E	C-S	25 (13/12/0)	2.8–5.8			19 (11/8/0)	2.8–5
35	Kisköre	River Tisza	30.04.2014	47°28′42.1″N 20°30′49.5″E	C-S	10 (6/4/0)	4.6–6				
36	Kisköre	River Tisza	30.06.2014	47°28′42.1″N 20°30′49.5″E	C-S	11 (7/4/0)	2.9–5.2				
37	Kisköre	River Tisza	30.08.2017	47°28′42.1″N 20°30′49.5″E	C-S	23 (11/12/0)	2.1–4.8				
38	Rózinowo	River Vistula	29.04.2016	52°43′21.8″N 18°59′11.1″E	C	14 (9/5/0)	2.9–5.6				
39	Rózinowo	River Vistula	04.11.2016	52°43′21.8″N 18°59′11.1″E	C	10 (3/5/2)	2–3.4				
40	Szolnok	River Zagyva	12.07.2016	47°10′24.5″N 20°12′09.4″E	C-S	24 (15/9/0)	2.2–5.2				

**Notes:**

Species codes: C, *C. curvispinum*, R, *C. robustum*, S, *C. sowinskyi*. Numbers refer to all individuals (females/males/juveniles) and min-max. body length.

* Included in Borza, Arbačiauskas & Zettler (2021).

** Included in Borza et al. (2018).

Geographic coordinates are approximate in most cases.

The specimens included in the analysis were selected from the samples to represent a body length range as broad as possible in both sexes. In most cases slightly more females were included since they grow larger. Small specimens (<~2 mm) without recognizable secondary sexual features were considered juveniles.

### Morphological measurements

**Figure 2:**
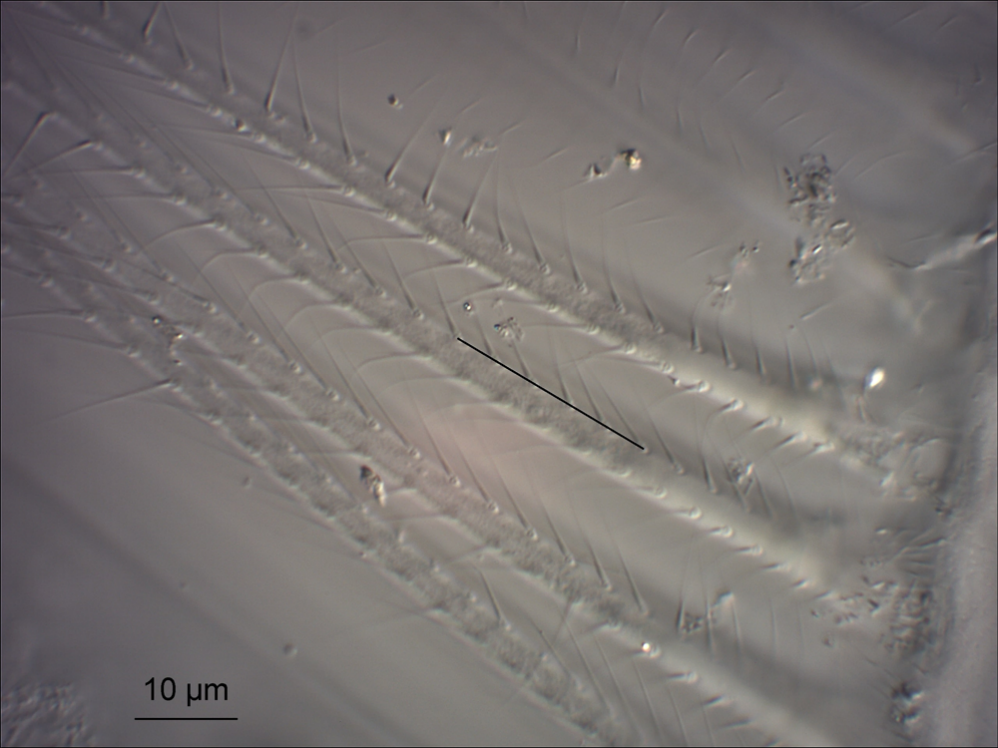
The filtering setae of *C. curvispinum* (4.2 mm, male). One of the original photographs used for the measurements. The black line illustrates the unit of measurement.

The morphological measurements were done conforming to the procedure described by Borza et al. (2018). After the measurement of standard body length (from the tip of the rostrum to the end of the telson; using ocular micrometer), microscopic preparations were made from the filtering setae. The part of the 2^nd^ gnathopods bearing the setae was dissected, mounted on a slide, and covered in Canada balsam. The measurements were made on digital photographs (Fig. 2) taken under light microscope (DIC, 1,000× magnification) using the ImageJ2 software (Rueden et al., 2017). To decrease measurement error, the distance between the centers of six bristles (spanning five gaps) near the basis of the setae was measured and the FMS was assessed by dividing the value of the measured distance by five. This procedure was repeated 10 times per specimen, each measurement performed on different setae. The full dataset is available under DOI: 10.6084/m9.figshare.12826535.

### Statistical analysis

The statistical analysis of the data was performed in R 3.6.3 (R Core Team, 2020). Two specimens (1 *C. curvispinum*, 1 *C. sowinskyi*) considered as outliers were excluded from the analysis (Fig. S1). In line with aim 1 (‘Range of intraspecific variation’), violin plots (‘vioplot’ package; Adler & Kelly, 2019) were used to visualize the overall range and distribution of individual FMS means per species. To allow the comparison among the species represented with different sample sizes, the individual-based rarefaction curves of the FMS ranges were generated using basic R functions.

In line with aim 2 (‘Components of intraspecific variation’), the within-individual variation of FMS was modelled per species with linear mixed-effect models (‘nlme’ package; Pinheiro et al., 2020), including individual FMS means, body length, and sex as fixed factors, and samples as a random factor (intercept). The marginal *R*^*2*^ (fixed effects) and conditional *R*^*2*^ (fixed and random effects) of the models (Nakagawa & Schielzeth, 2013) was calculated using the ‘sem.model.fits’ function (‘piecewiseSEM’ package; Lefcheck, 2016).

To reveal the components of among-individual variation, linear mixed-effect models were used for samples containing more than 18 specimens of a species without juveniles and a male/female sex ratio higher than 2/3 (*N* = 22 for *C. curvispinum*, *N* = 6 for *C. robustum*, and *N* = 8 for *C. sowinskyi*). The selection of the optimal models was based on the protocol proposed by (Zuur et al., 2009). Individuals were considered as a random effect (intercept) in all cases. The optimal variance structures were selected in models including all considered fixed effects. The set of potential variance covariates and functions was based on the results of the modelling of within-individual variation. The variance structure with the lowest Akaike Information Criterion (*AIC*) was considered as optimal, if the likelihood-ratio test with the second best model was significant. Otherwise, the simpler (fewer degrees of freedom) model was selected. Body length (first or second order polynomial) and sexes were considered as fixed effects with or without interaction, implying eight potential combinations. The selection of the optimal combination of fixed effects was based on the likelihood-ratio test (stepwise elimination of non-significant effects starting with the most complex model) using models fitted with the maximum likelihood (‘ML’) method. The optimal models were refitted with the restricted maximum likelihood (‘REML’) method for the estimation of parameters and variance components.

To characterize the component of among-individual variation not explainable by the body length and sex effects (i.e., random effect), models with the most complex fixed formula (2^nd^ order polynomial of body length in interaction with sex) without variance covariates were fitted per samples and species. The relative share of variance components (fixed, random, and residual) was visualized in two-dimensional scatterplots.

In line with aim 3 (‘Drivers of intraspecific variation’), the effect of variables potentially accounting for the variation among samples was tested in mixed-effect models. The simultaneous analysis of all available explanatory variables was not feasible due to the heterogeneity of the material. The effect of species combinations could only be tested in *C. curvispinum* and *C. sowinskyi*, since *C. robustum* co-occured with the other two species in all samples. In these models, mean-centered FMS data were used as the dependent variable, the presence/absence of congeneric competitors was included as the fixed effect, the random effect comprised three nested levels: (1) water body (as in Table 1), (2) sample and body length, and (3) individual (body length was included as a random slope while the other terms as random intercepts), whereas individual FMS means and body length were used as variance covariates (power function). The FMS differences between species combinations were estimated by Tukey contrasts using the ’multcomp’ package (Hothorn, Bretz & Westfall, 2008).

The potential effect of habitat types could be tested based on a rough categorization (stagnant vs. flowing waters) only in *C. curvispinum*, since only this species occurred in stagnant waters (in the Baltic Sea and Lake Balaton; samples 1–7). However, habitat types could not be analyzed jointly with species combinations due to the large overlap in the two factors (only samples 38–39 from the River Vistula represented allopatric occurrences of the species in a river). Therefore, the habitat effect was tested separately in a similar model as species combinations (Tukey contrasts were not necessary in this case since two types were considered only), and the potential roles of the two effects are discussed.

Temporal differences in FMS could be tested directly in samples taken at different times at the same site. Within-year changes (samples 33–34, 35–36, and 38–39 for *C. curvispinum*, and 33-34 for *C. sowinskyi*) were analyzed in mixed-effect models featuring sample IDs, sex, body length, and all their interactions as fixed effects, individual IDs as a random effect, and individual FMS means and body length as variance covariates (power function). The optimal combination of fixed effects was determined based on likelihood-ratio tests in ‘ML’ models, and the parameters were estimated in the optimal model refitted with ‘REML’. Among-year differences were tested with a similar approach in samples 33-37 for *C. curvispinum*, in this case including years as a fixed effect instead of sample IDs.

In line with aim 4 (‘Consequences on niche differentiation’), interspecific differences were characterized based on the FMS range overlaps in co-occurring populations.

## Results

### Range of intraspecific variation

**Figure 3:**
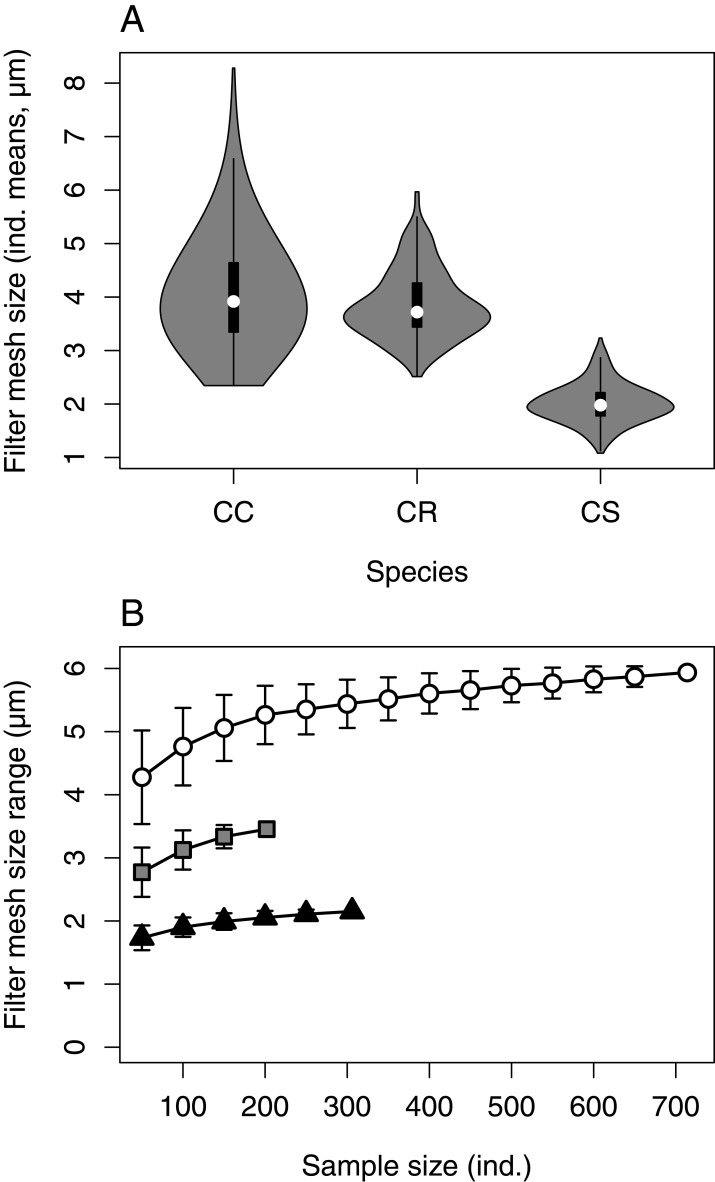
FMS means and ranges by species. (A) The distribution of individual FMS means in the studied material per species. CC, *C. curvispinum*; CR, *C. robustum*; CS, *C. sowinskyi*. (B) FMS ranges (±*SD*) as a function of sample size (individual-based rarefaction curves). White circles: *C. curvispinum*, grey squares: *C. robustum*, black triangles: *C. sowinskyi*.

Individual FMS means ranged in the studied material between 2.34 and 8.28 μm in *C. curvispinum*, between 2.51 and 5.97 μm in *C. robustum*, and between 1.08 and 3.23 μm in *C. sowinskyi* (Fig. 3A). The individual-based rarefaction curves indicated that the differences in FMS ranges among the three species were largely independent of sample sizes (Fig. 3B).

### Components of intraspecific variation

In all three species, the *ln*-transformed within-individual variation of FMS showed linear positive dependence on the *ln*-transformed individual mean of FMS and linear negative dependence on the ln-transformed body length, whereas sexes did not have a significant effect (Table 2). The random effect increased the *R*^*2*^ of the models only moderately, indicating little differences in the within-individual variation of FMS among samples (Table 2).

**Table 2:** Parameter estimates (fixed effects) and explained variance proportions of the mixed-effect models of within-individual variation. All *p* < 0.0001.

Species	Intercept		FMS mean (*ln*)		Body length (*ln*)		Marginal *R*^*2*^	Conditional *R*^*2*^
	Estimate	*SE*	Estimate	*SE*	Estimate	*SE*		
*C. curvispinum* (*df* = 697)	−6.29	0.18	3.21	0.14	−0.52	0.10	0.54	0.57
*C. robustum* (*df* = 190)	−5.53	0.43	3.98	0.37	−1.42	0.18	0.40	0.43
*C. sowinskyi* (*df* = 287)	−5.20	0.19	3.53	0.30	−1.04	0.19	0.37	0.41

The optimal models per samples and species included various combinations of fixed effects and variance covariates (Table 3, Table S1–S3, Fig. S2), indicating that the sources of among-individual variation of FMS were variable among populations. Seven out of the eight possible combinations of fixed effects was observed with the exception of non-linear body length effect without sex effect (row 3 in Table 3). The lack of both body length and sex effects (row 1 in Table 3) was relatively common in *C. curvispinum* and *C. sowinskyi*; however, it was not observed in *C. robustum*. The sex effect occurred almost always coupled with a certain type of body length effect (with or without interaction; rows 5–8 in Table 3) with only one exception in *C. robustum*. Non-linear body length effect (rows 6 and 8 in Table 3) was observed in *C. curvispinum* and *C. robustum* but not in *C. sowinskyi*.

**Table 3:** The number of samples with different combinations of variance covariates (columns) and fixed effects (rows) in the optimal mixed effect models of FMS.

	*C. curvispinum*				*C. robustum*				*C. sowinskyi*			
	None	L	M	LM	None	L	M	LM	None	L	M	LM
(intercept)	2	1	3	1							3	
L1	1		3	1				2				
L2												
S								1				
L1+S			3	1					2		1	
L2+S			1									
L1*S			2					2				2
L2*S	1		1	1				1				

**Notes:**

L, body length; M, mean FMS; S, sex; 1, first power; 2, second power; +, without interaction; *, with interaction.

**Figure 4:**
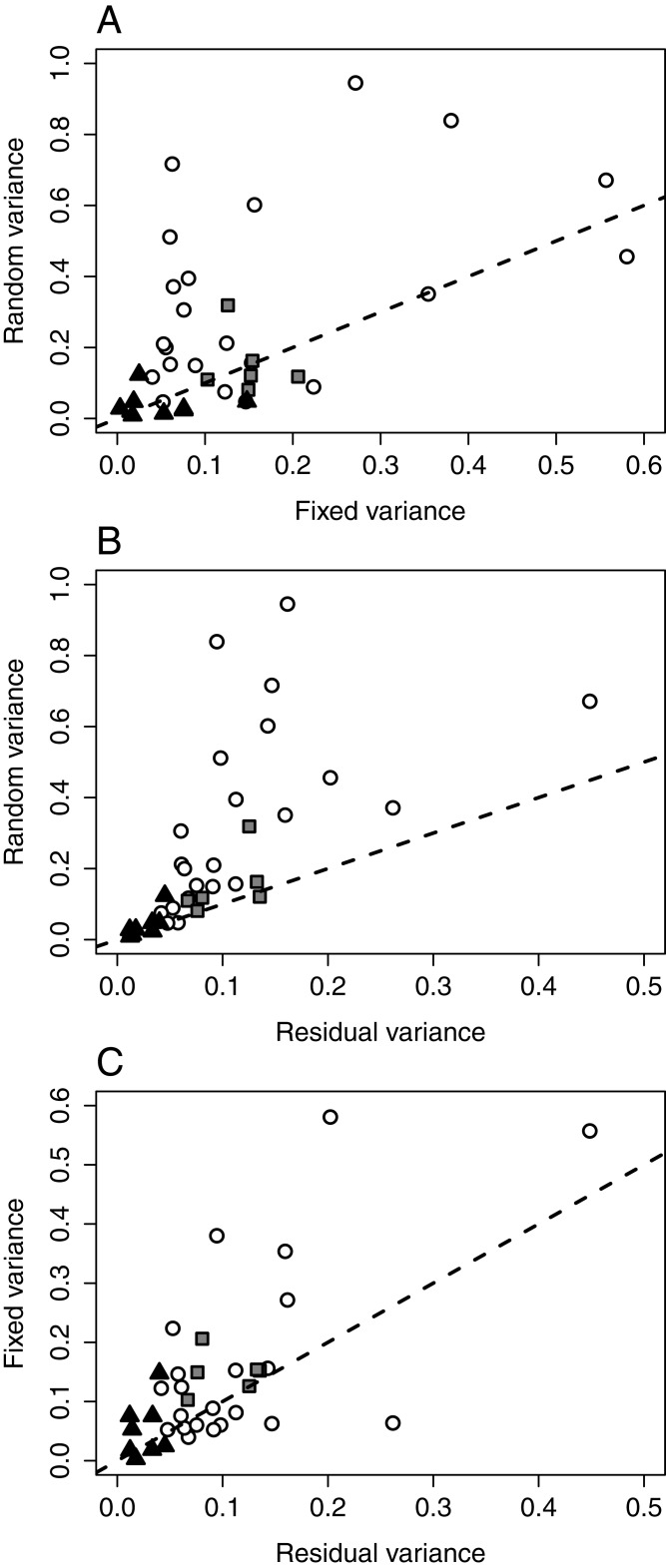
Variance components in the mixed-effect models of FMS by samples and species (all fixed effects included). (A) Fixed vs. random. (B) Residual vs. random. (B) Residual vs. fixed. White circles: *C. curvispinum*, grey squares: *C. robustum*, black triangles: *C. sowinskyi*. Dashed lines indicate slope = 1.

The models featuring all fixed effects (allowing the comparison of the fixed, random, and residual components of among-individual variation within samples) revealed that random variance among individuals exceeded the component explained by all considered fixed factors in the majority of samples in *C. curvispinum* (16 out of 22), but not in *C. robustum* (3 out of 6) or *C. sowinskyi* (3 out of 8; Fig. 4A, Table S4). The random component also exceeded residual (i.e., within-individual) variance in most cases in all three species (20 out of 22 in *C. curvispinum*, 5 out of 6 in *C. robustum*, and 6 out of 8 in *C. sowinskyi*; Fig. 4B, Table S4). The variance explained by the fixed effects exceeded residual variance in 13 out of 22, 6 out of 6, and 5 out of 8 cases in *C. curvispinum*, *C. robustum*, and *C. sowinskyi*, respectively (Fig. 4C, Table S4).

### Drivers of intraspecific variation

**Figure 5:**
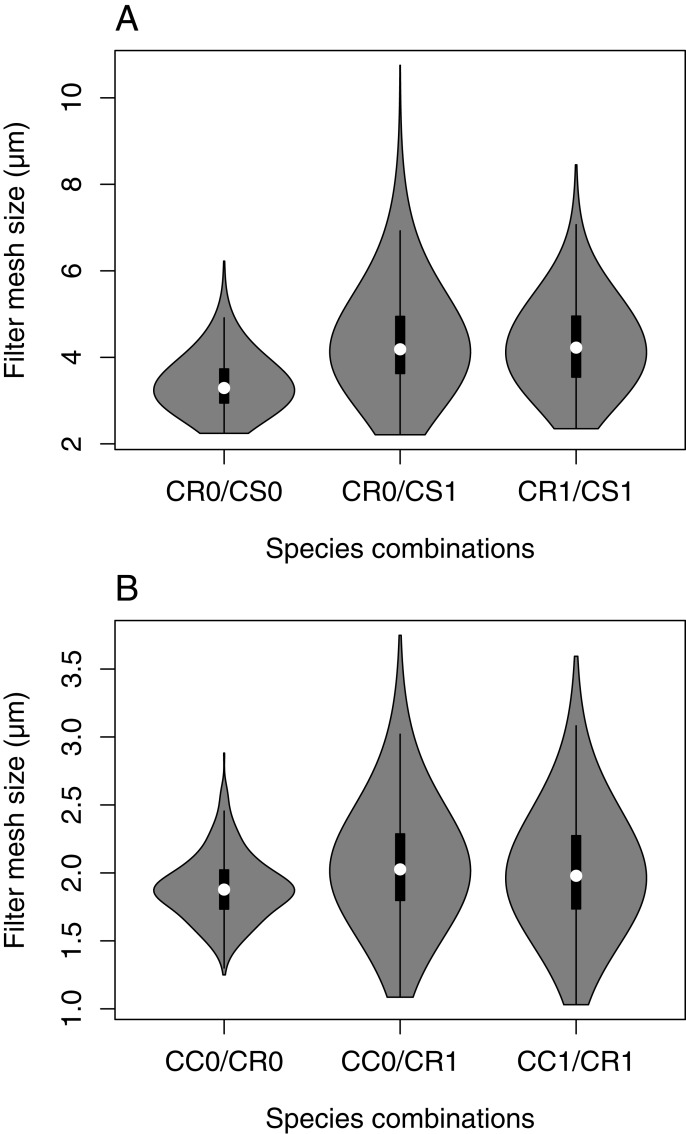
FMS as a function of the presence/absence of congeneric competitors. (A) *C. curvispinum*. (B) *C. sowinskyi*. CC, *C. curvispinum*; CR, *C. robustum*; CS, *C. sowinskyi*. 0, absent; 1, present.

The mixed-effect model including species combinations as an explanatory variable indicated that the FMS of *C. curvispinum* was significantly smaller in allopatry (mean ± *SE*: 3.41 ± 0.25 μm at 3.75 mm body length; Fig. 5A, Table 4) than in the presence of *C. sowinskyi* only (4.31 ± 0.19 μm). However, the presence of *C. robustum* did not increase the FMS of *C. curvispinum* any further (3.99 ± 0.20 μm). The presence or absence of congeneric competitors did not have a significant effect on the FMS of *C. sowinskyi* (1.84 ± 0.10 μm alone, 2.01 ± 0.08 μm in the presence of *C. curvispinum* only, and 1.96 ± 0.09 μm in the presence of both other species at 3.55 mm body length; Fig. 5B, Table 4).

**Table 4:** Parameter estimations of the mixed-effect models testing the effect of different variables on FMS.

Tested effect	Species	Samples	Parameter contrast	Estimate	*SE*	*p*
Presence/absence of competitors	CC	all	‘CR0/CS0’–‘CR0/CS1’	−0.89	0.32	0.0134*
			‘CR0/CS0’–‘CR1/CS1’	−0.57	0.32	0.1693
			‘CR0/CS1’–‘CR1/CS1’	0.32	0.20	0.2438
	CS	all	‘CC0/CR0’–‘CC1/CR0’	−0.17	0.10	0.2160
			‘CC0/CR0’–‘CC1/CR1’	−0.12	0.10	0.4490
			‘CC1/CR0’–‘CC1/CR1’	0.05	0.09	0.8430
Habitat type	CC	all	‘Stagnant’-‘Flowing’	−0.86	0.31	0.0204*
Temporal change within a year	CC	River Tisza, 2013 (33, 34)	‘31 July’–‘14 May’	−0.42	0.27	0.1356
	CC	River Tisza, 2014 (35, 36)	‘30 June’–‘30 April’	0.14	0.37	0.7058
	CS	River Tisza, 2013 (33, 34)	‘31 July’–‘14 May’	−0.37	0.10	0.0001*
	CC	River Vistula (38, 39)	‘4 November’–‘29 April’	−0.60	0.22	0.0138*
Temporal change among years	CC	River Tisza, 2013–2017 (33–37)	‘2014’–‘2013’	0.01	0.16	0.9987
			‘2017’–‘2013’	−0.49	0.18	0.0177*
			‘2017’–‘2014’	−0.50	0.21	0.0451*

**Notes:**

CC, *C. curvispinum;* CR, *C. robustum*; CS, *C. sowinskyi*; 0, absent; 1, present.

* *p* < 0.05.

The model involving habitat types estimated a similar FMS difference in *C. curvispinum* between stagnant and flowing waters as between the species combinations (alone vs. with *C. sowinskyi*; Table 4).

The modeling of temporal differences revealed that within-year changes in the FMS of *C. curvispinum* in the River Tisza (Kisköre) could be explained by sex and body length effects (Table 4, Fig. S3). By contrast, a significant temporal effect was detected in *C. sowinskyi* at the same site in the year 2013 (Table 4, Fig. S4). Similarly, the seasonal difference as well as the body length effect was significant in *C. curvispinum* in the River Vistula (Table 4, Fig. S5).

The modeling of all five samples from Kisköre revealed that the sex and body length effects were similar in all three years (i.e., the interactions were not significant), making the estimation of among-year differences straightforward. The FMS of *C. curvispinum* did not change significantly between 2013–2014; however, the decrease by 2017 was significant compared to both previous years (Table 4, Fig. S3).

### Consequences on niche differentiation

**Figure 6:**
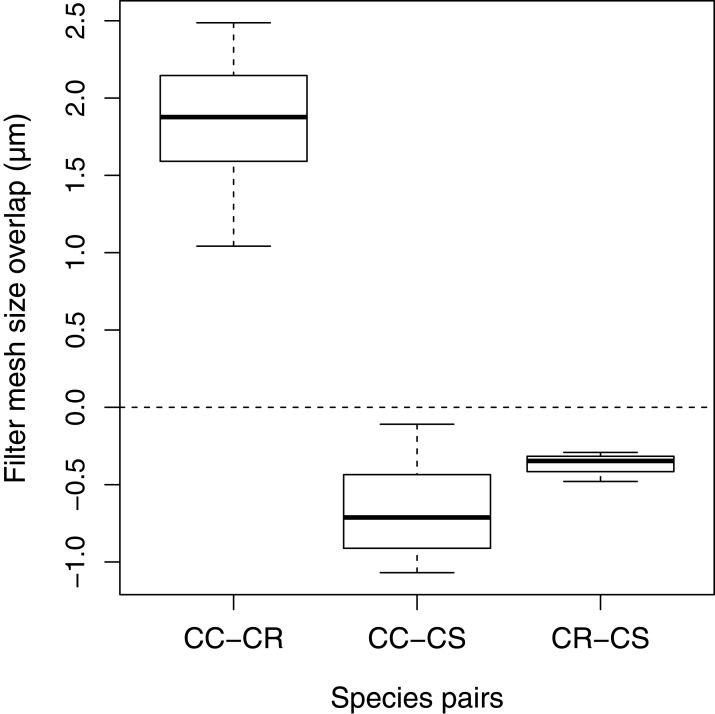
Overlaps in the FMS range of the species in co-occurrence. CC, *C. curvispinum*; CR, *C. robustum*; CS, *C. sowinskyi*.

The FMS ranges of *C. curvispinum* and *C. robustum* overlapped considerably in all samples where they were both present (mean ± *SD*: 1.88 ± 0.48 μm, *N* = 9; Fig. 6). By contrast, the FMS ranges of *C. curvispinum* as well as *C. robustum* never overlapped with that of *C. sowinskyi* (−0.67 ± 0.30 μm, *N* = 10 between *C. curvispinum* and *C. sowinskyi*; −0.37 ± 0.08 μm, *N* = 4 between *C. robustum* and *C. sowinskyi*; Fig. 6).

## Discussion

### Range of intraspecific variation

The analysis revealed that FMS in corophiids shows considerable and complex variability; however, the extent of the variation might be different per species. Among the three studied *Chelicorophium* sp., the FMS of the most successful invader–*C. curvispinum*–varied within the broadest range, indicating that the trait is more flexible in this species than in the other two. Although the data do not prove that the other two species would not be able to shift their FMS beyond the observed limits under certain circumstances, the rarefaction indicates that the difference is not a mere sampling artefact. This was also supported by the fact that the FMS of *C. curvispinum* was highly variable even locally in some of the samples. The relationship between trait plasticity and invasion success is equivocal; a meta-analysis on plants concluded that invasive species are on average not more plastic than their native or non-invasive counterparts (Palacio-López & Gianoli, 2011). The higher invasion success of *C. curvispinum* compared to the other two species is attributable mainly to the fact that it was the only species that could expand its range in the central invasion corridor (Bij de Vaate et al., 2002) which might have several different explanations. Being able to adjust the FMS within a broad range appears to be an inherently advantageous skill; however, how much this might have contributed to the invasion success of the species is hard to tell. Nevertheless, it would also be interesting to compare the three invasive Ponto-Caspian species with the non-invasive ones in this regard.

### Components of intraspecific variation

The within-individual variation of FMS was primarily determined by the individual mean of the trait. Since variable mesh sizes within a net are not adaptive (Crittenden, 1981), this might indicate that the reliability of the morphogenesis decreases as the distances between the bristles increase, which in the end might determine the upper limit of FMS within the group. The within-individual variability of FMS also decreased slightly with body size which might indicate selection during the ontogenesis where individuals with lower variability have a higher chance of attaining large body size. Developmental instability often reflects environmental stress (De Anna, Bonisoli-Alquati & Mousseau, 2013); however, since the within-individual variation of FMS showed little differences among samples, such a relationship seems to be unlikely, at least within the stress gradient represented by the studied samples.

The analysis revealed that the among-individual variation of FMS within populations could be partitioned into body size and sex-related as well as seemingly random components, the absolute extent and relative contribution of which varied within wide limits. The rather heterogeneous material of this explorative study did not allow variance components to be used as dependent variables; nevertheless, they might be indicative of important ecological phenomena and so are worthy of further investigation.

The body length dependency of FMS has been observed previously in corophiids as well as in other crustacean taxa (Brendelberger & Geller, 1985; Suh & Choi, 1998). This pattern most likely reflects an ontogenetic niche shift induced by intraspecific competition (Nakazawa, 2015); however, differences among cohorts cannot be excluded either, especially if the relationship is not linear as in some of the samples in the present study. Borza, Arbačiauskas & Zettler (2021) found that the effect was missing in *Corophium multisetosum* Stock, 1952 in the Baltic Sea and the present results showed that the extent of body size dependency can vary within species, as well. The strength of the relationship might depend on the intensity of intraspecific competition modulated by the abundance and/or size distribution of food particles.

Borza et al. (2018) supposed that the slight intersexual differences observed the Lower Danube in the three invasive Ponto-Caspian *Chelicorophium* species might be related to the high overall dimorphism in the group affecting the body size measurement, so the phenomenon is ecologically not relevant. However, in the light of the present results showing that sex-related differences vary among samples, it seems more likely that they indeed have a relevant biological background. Sexual dimorphism in niche-related traits can evolve as a result of the interplay between sexual selection and ecological character displacement (De Lisle, 2019). Remarkably, the sex effect was accompanied by body length dependency in almost all samples, indicating that it might also be related to the intensity of intraspecific competition. However, the degree of sexual dimorphism as well as its interaction with body size was variable among the samples exhibiting body length dependency in FMS, implying that the two effects are not regulated completely in the same way.

The unexplained component of among-individual variation can be interpreted as individual specialization, usually driven by intra- and interspecific competition, ecological opportunity, and predation (Bolnick et al., 2003; Araújo, Bolnick & Layman, 2011; Dall et al., 2012). Given that corophiids are sedentary animals, a potential determinant in the present case might be the micro-scale position of individuals, influencing their food supply through the density and species composition of their neighbors; i.e., the strength of intra- and interspecific competition (Tilman, 1994). Since the literature on individual specialization is based predominantly on behavioral traits or food composition, studying the morphological variability of filtering structures in corophiids might provide important contributions to our general understanding of the phenomenon.

### Drivers of intraspecific variation

The analysis revealed an apparent pattern in the FMS of *C. curvispinum* in relation to species combinations, namely that the species had consistently dense filters when occurring alone, while it had on averages sparser but highly variable filters in the presence of *C. sowinskyi* (irrespective of the presence of *C. robustum*). Nevertheless, since two out of the three waters with allopatric occurrence were stagnant (Baltic Sea, Lake Balaton), and the FMS values of the species third, riverine site (River Vistula) did not represent an outlier compared to the sympatric samples, habitat types (stagnant vs. flowing) could explain the pattern with similar probability.

The competitor-effect seems plausible, since the two species show marked differences in FMS indicating niche differentiation by food particle size; therefore, the shift of *C. curvispinum* towards larger FMS in the presence of *C. sowinskyi* could be interpreted as ecological character displacement (Dayan & Simberloff, 2005). Nevertheless, some of the sympatric samples show that the coexistence of the two species is possible even with FMS in *C. curvispinum* as small as in the River Vistula. Also, parallel changes could not be observed in *C. sowinskyi*; however, this might potentially be attributable to the asymmetric nature of the interaction; i.e., smaller mesh sizes can capture large particles but not vice versa, implying that the intensity of competition is stronger on the species with the coarser mesh.

Current velocity has been demonstrated to regulate FMS in hydropsychid caddisflies (Loudon & Alstad, 1992); however, its direct effect in the present case seems unlikely. Corophiids are active filterers, creating currents inside their tubes by the beating of their pleopods; therefore, they do not depend on the ambient currents. In the end, competitors and currents both can influence the abundance and size distribution of food particles; therefore, they can be expected to have an indirect effect on FMS. However; disentangling their roles would require more detailed data on the suspended matter.

The inconsistent results on temporal changes might have a similar explanation. The size distribution of suspended particles might shift variably in time, resulting in different pressures for FMS adaptation. Nevertheless, the fact that significant FMS shifts have been observed in some cases at a time interval of only ~2.5 months provides some insight into the mechanism of the adaptation. Although this interval is commensurate with the generation time of corophiids (Muskó, 1992) allowing changes to occur even in genetically determined traits by natural selection, such a consistent shift between two generations indicates the dominant role of phenotypic plasticity.

### Consequences on niche differentiation

Information on the intraspecific variation of FMS put the interactions among the species into a new perspective (Borza et al., 2018). The fact that the FMS ranges of *C. curvispinum* and *C. sowinskyi* never overlapped in co-occurrence despite the considerable intraspecific differences among sites confirmed that their interaction can be seen as a clear case of niche differentiation by food particle size. The consistent differences between the FMS ranges of *C. robustum* and *C. sowinskyi* suggest that their interaction is similar to the one between *C. curvispinum* and *C. sowinskyi*, although somewhat less flexible. However, the strong overlaps and in some cases the almost complete absence of differentiation between *C. curvispinum* and *C. robustum* calls the role of food particle size as the most important niche axis allowing their coexistence into question. Although the potential for differentiation by FMS might increase the stability of their coexistence (Ashby et al., 2017), other factors–possibly related to body size differences (Borza et al., 2018)–can be assumed to play a decisive role, as well.

## Conclusions

In summary, the study revealed a considerable extent of intraspecific variation in the FMS of invasive Ponto-Caspian corophiids which might potentially be reflected in the food web positions of the species. The identified components of variation were themselves variable among populations, indicating that intra- and interspecific competition can modulate the FMS in complex ways. The results also contributed to our understanding of the niche differentiation among the species; however, the heterogeneity of the material allowed only a limited insight into the drivers and mechanisms of variability, warranting further studies with more strictly controlled field parameters and experimental approaches.

Although there might be idiosyncrasies among groups of suspension feeders differing in filtering mechanisms, habitat use, and life history; intraspecific variability in FMS can be expected to be widespread and potentially be of community-level importance in keystone species. Identifying the drivers and mechanisms of the variability might lead to a better understanding of the functioning of aquatic ecosystems as well as a better ability at assessing and predicting the impacts of anthropogenic disturbances. The studied species–especially the most flexible *C. curvispinum*–appear to be suitable model organisms to this end.

## Supplemental Information

10.7717/peerj.11245/supp-1 Supplemental Information 1 FMS (individual means ± *SD*) in the samples containing the specimens considered as ouliers. (A) *C. curvispinum* in Sample no. 8 (as in Table 1). (B) *C. sowinskyi* in Sample no. 33. Circles: females, triangles: males. The individuals considered outliers are marked red. The FMS of the individual in (A) is outstanding only in the sample. This sampling site was under the influence of a small tributary; the presence of the outlier might perhaps be a result of drift from a population under different selection pressures. The individual in (B) is a global outlier in the studied material of *C. sowinskyi*. Its unusually large FMS might potentially be a result of developmental abnormality.

10.7717/peerj.11245/supp-2 Supplemental Information 2 FMS (individual means ± *SD*) per species and samples (as in Table 1). CC: *C. curvispinum*, CR: *C. robustum*, CS: *C. sowinskyi*. Red circles: females, blue triangles: males. The lines indicate the fixed effects of the optimal mixed-effect models (as defined in the text; Table S1-S2-S3).

10.7717/peerj.11245/supp-3 Supplemental Information 3 The FMS (individual means ± *SD*) of *C. curvispinum* in samples no. 33-37 (collected at the same site at different times in the River Tisza). Colors indicate the years of collection; black: 2013 (empty symbols: sample no. 33, filled symbols: 34), red: 2014 (empty symbols: 35, filled symbols: 36), blue: 2017. Circles: females, triangles: males. The lines of corresponding colors represent the fixed effects (year and body length; the interaction was not significant) of the model including all five samples. The Tukey’s post-hoc test indicated significant differences between 2013- 2017 (estimate ± *SE*: -0.70 ± 0.22 μm, *p* = 0.0034), and 2014- 2017 (-0.64 ± 0.26 μm, *p* = 0.0317), but not between 2013-2014 (-0.06 ± 0.19 μm, *p* = 0.9473). Within-year differences (tested in separate models for 2013 and 2014) were explained by body length and sexes (2013: sex_male: 1.01 ± 0.22 μm, *p* < 0.001; body length: 0.63 ± 0.12 μm, *p* < 0.001; 2014: sex_male: 1.14 ± 0.24 μm, *p* = 0.0002; body length: 0.46 ± 0.16 μm, *p* = 0.0086).

10.7717/peerj.11245/supp-4 Supplemental Information 4 The FMS (individual means ± *SD*) of *C. sowinskyi* in samples no. 33-34, collected at the same site in the River Tisza at different times within a year. White: no. 33, 14.05.2013, black: no. 34, 31.07.2013. Circles: females, triangles: males. The lines represent the fixed effects of the optimal mixed-effect model including both samples. The difference between the samples was significant (mean ± *SE*: -0.37 ± 0.10, *p* = 0.0001).

10.7717/peerj.11245/supp-5 Supplemental Information 5 The FMS (individual means ± *SD*) of *C. curvispinum* in samples no. 38-39, collected at the same site in the River Vistula at different times within a year. White: no. 38, 29.04.2016, black: no. 39, 04.11.2016. Circles: females, triangles: males, squares: juveniles. The lines represent the fixed effects of the optimal mixed-effect model including both samples. The body length effect (mean ± *SE*: 0.42 ± 0.10, *p* = 0.0001) and the difference between the samples (mean ± *SE*: -0.60 ± 0.22, *p* = 0.0138) were significant.

10.7717/peerj.11245/supp-6 Supplemental Information 6 *AIC* values and the *p*-values of the likelihood-ratio tests. Akaike Information Criterion (*AIC*) values and the *p*-values of likelihood-ratio tests (comparing the models with the lowest and second lowest, the second and third lowest, and the third and fourth lowest *AIC*) serving as the basis for the selection of optimal variance structures (weights) in the mixed-effect models per species and samples (see the text for explanation).

10.7717/peerj.11245/supp-7 Supplemental Information 7 The *p*-values of the likelihood-ratio tests serving as the basis for the selection of the optimal combination of fixed effects in the mixed-effect models per species and samples (see the text for explanation). L: body length, S: sex, 1: first power, 2: second power, +: without interaction, *: with interaction.

10.7717/peerj.11245/supp-8 Supplemental Information 8 Variance components, weights, and parameter estimations of the optimal mixed-effect models per species and samples. L: body length, S: sex, 1: first power, 2: second power, ’:’ : interaction.

10.7717/peerj.11245/supp-9 Supplemental Information 9 Variance components of the mixed-effect models including all fixed effects.

## Additional Information and Declarations

### Data Availability

The following information was supplied regarding data availability: The full dataset is available under DOI: 10.6084/m9.figshare.12826535.

## References

[ref-1] Abonyi A, Ács É, Hidas A, Grigorszky I, Várbíró G, Borics G, Kiss KT (2018). Functional diversity of phytoplankton highlights long-term gradual regime shift in the middle section of the Danube River due to global warming, human impacts and oligotrophication. Freshwater Biology.

[ref-2] Abonyi A, Kiss KT, Hidas A, Borics G, Várbíró G, Ács É (2020). Cell size decrease and altered size structure of phytoplankton constrain ecosystem functioning in the middle danube river over multiple decades. Ecosystems.

[ref-3] Adler D, Kelly ST (2019). Vioplot: violin plot. R package version 0.3.4. https://github.com/TomKellyGenetics/vioplot.

[ref-4] Alstad DN (1987). Particle size, resource concentration, and the distribution of net-spinning caddisflies. Oecologia.

[ref-5] Araújo MS, Bolnick DI, Layman CA (2011). The ecological causes of individual specialisation. Ecology Letters.

[ref-6] Ashby B, Watkins E, Lourenço J, Gupta S, Foster KR (2017). Competing species leave many potential niches unfilled. Nature Ecology & Evolution.

[ref-7] Atkinson A, Hill SL, Barange M, Pakhomov EA, Raubenheimer D, Schmidt K, Simpson SJ, Reiss C (2014). Sardine cycles, krill declines, and locust plagues: revisiting ‘wasp-waist’food webs. Trends in Ecology & Evolution.

[ref-8] Atkinson CL, Vaughn CC, Forshay KJ, Cooper JT (2013). Aggregated filter-feeding consumers alter nutrient limitation: consequences for ecosystem and community dynamics. Ecology.

[ref-9] Barnett TP, Adam JC, Lettenmaier DP (2005). Potential impacts of a warming climate on water availability in snow-dominated regions. Nature.

[ref-10] Bij de Vaate AB, Jażdżewski K, Ketelaars HAM, Gollasch S, Van der Velde G (2002). Geographical patterns in range extension of Ponto-Caspian macroinvertebrate species in Europe. Canadian Journal of Fisheries and Aquatic Sciences.

[ref-11] Bolnick DI, Amarasekare P, Araújo MS, Bürger R, Levine JM, Novak M, Rudolf VH, Schreiber SJ, Urban MC, Vasseur DA (2011). Why intraspecific trait variation matters in community ecology. Trends in Ecology & Evolution.

[ref-12] Bolnick DI, Svanbäck R, Fordyce JA, Yang LH, Davis JM, Hulsey CD, Forister ML (2003). The ecology of individuals: incidence and implications of individual specialization. The American Naturalist.

[ref-13] Borza P, Arbačiauskas K, Zettler ML (2021). Multidimensional niche differentiation might buffer invasion impacts: the case of oligohaline corophiids (Crustacea: Amphipoda) in the Baltic Sea. Biological Invasions.

[ref-14] Borza P, Csányi B, Huber T, Leitner P, Paunović M, Remund N, Szekeres J, Graf W (2015). Longitudinal distributional patterns of Peracarida (Crustacea, Malacostraca) in the River Danube. Fundamental and Applied Limnology.

[ref-15] Borza P, Huber T, Leitner P, Remund N, Graf W (2018). Niche differentiation among invasive Ponto-Caspian *Chelicorophium* species (Crustacea, Amphipoda, Corophiidae) by food particle size. Aquatic Ecology.

[ref-16] Brendelberger H, Geller W (1985). Variability of filter structures in eight *Daphnia* species: mesh sizes and filtering areas. Journal of Plankton Research.

[ref-17] Crittenden RN (1981). Morphological characteristics and dimensions of the filter structures from three species of *Daphnia* (Cladocera). Crustaceana.

[ref-18] Dall SR, Bell AM, Bolnick DI, Ratnieks FL (2012). An evolutionary ecology of individual differences. Ecology Letters.

[ref-19] Dayan T, Simberloff D (2005). Ecological and community-wide character displacement: the next generation. Ecology Letters.

[ref-20] De Anna EB, Bonisoli-Alquati A, Mousseau TA (2013). The use of fluctuating asymmetry as a measure of environmentally induced developmental instability: a meta-analysis. Ecological Indicators.

[ref-21] De Lisle SP (2019). Understanding the evolution of ecological sex differences: Integrating character displacement and the Darwin-Bateman paradigm. Evolution Letters.

[ref-22] Des Roches S, Post DM, Turley NE, Bailey JK, Hendry AP, Kinnison MT, Schweitzer JA, Palkovacs EP (2018). The ecological importance of intraspecific variation. Nature Ecology & Evolution.

[ref-23] Gerdol V, Hughes RG (1994). Effect of *Corophium volutator* on the abundance of benthic diatoms, bacteria and sediment stability in two estuaries in southeastern England. Marine Ecology Progress Series.

[ref-24] Germanov ES, Marshall AD, Bejder L, Fossi MC, Loneragan NR (2018). Microplastics: no small problem for filter-feeding megafauna. Trends in Ecology & Evolution.

[ref-25] Higgins SN, Vander Zanden MJ (2010). What a difference a species makes: a meta-analysis of dreissenid mussel impacts on freshwater ecosystems. Ecological Monographs.

[ref-26] Hothorn T, Bretz F, Westfall P (2008). Simultaneous inference in general parametric models. Biometrical Journal.

[ref-27] Jourdan J, Piro K, Weigand A, Plath M (2019). Small-scale phenotypic differentiation along complex stream gradients in a non-native amphipod. Frontiers in Zoology.

[ref-28] Kang C-K, Choy EJ, Hur Y-B, Myeong J-I (2009). Isotopic evidence of particle size-dependent food partitioning in cocultured sea squirt *Halocynthia roretzi* and Pacific oyster *Crassostrea gigas*. Aquatic Biology.

[ref-29] Lampert W, Brendelberger H (1996). Strategies of phenotypic low-food adaptation in *Daphnia*: filter screens, mesh sizes, and appendage beat rates. Limnology and Oceanography.

[ref-30] Lefcheck JS (2016). PiecewiseSEM: piecewise structural equation modelling in R for ecology, evolution, and systematics. Methods in Ecology and Evolution.

[ref-31] Lewandowska AM, Boyce DG, Hofmann M, Matthiessen B, Sommer U, Worm B (2014). Effects of sea surface warming on marine plankton. Ecology Letters.

[ref-32] Loudon C, Alstad DN (1992). Architectural plasticity in net construction by individual caddisfly larvae (Trichoptera: Hydropsychidae). Canadian Journal of Zoology.

[ref-33] Muskó IB, Ilmavirta V, Jones RI (1992). Life history of Corophium curvispinum G. O. Sars (Crustacea, Amphipoda) living on macrophytes in Lake Balaton. The Dynamics and Use of Lacustrine Ecosystems. Developments in Hydrobiology.

[ref-34] Nakagawa S, Schielzeth H (2013). A general and simple method for obtaining R^2^ from generalized linear mixed-effects models. Methods in Ecology and Evolution.

[ref-35] Nakazawa T (2015). Ontogenetic niche shifts matter in community ecology: a review and future perspectives. Population Ecology.

[ref-36] Palacio-López K, Gianoli E (2011). Invasive plants do not display greater phenotypic plasticity than their native or non-invasive counterparts: a meta-analysis. Oikos.

[ref-37] Pinheiro J, Bates D, DebRoy S, Sarkar D, R Core Team (2020). Nlme: linear and nonlinear mixed effects models. R package version 3.1-144. http://CRAN.R-project.org/package=nlme.

[ref-38] R Core Team (2020). R: a language and environment for statistical computing.

[ref-39] Riisgård HU, Larsen PS (2010). Particle capture mechanisms in suspension-feeding invertebrates. Marine Ecology Progress Series.

[ref-40] Rueden CT, Schindelin J, Hiner MC, DeZonia BE, Walter AE, Arena ET, Eliceiri KW (2017). ImageJ2: imageJ for the next generation of scientific image data. BMC Bioinformatics.

[ref-41] Santi F, Riesch R, Baier J, Grote M, Hornung S, Jüngling H, Plath M, Jourdan J (2020). A century later: adaptive plasticity and rapid evolution contribute to geographic variation in invasive mosquitofish. Science of the Total Environment.

[ref-42] Suh H-L, Choi S-D (1998). Comparative morphology of the feeding basket of five species of *Euphausia* (Crustacea, Euphausiacea) in the western North Pacific, with some ecological considerations. Hydrobiologia.

[ref-43] Tilman D (1994). Competition and biodiversity in spatially structured habitats. Ecology.

[ref-44] Violle C, Enquist BJ, McGill BJ, Jiang LIN, Albert CH, Hulshof C, Jung V, Messier J (2012). The return of the variance: intraspecific variability in community ecology. Trends in Ecology & Evolution.

[ref-45] Zuur AF, Ieno EN, Walker NJ, Saveliev AA, Smith GM (2009). Mixed effects models and extensions in ecology with R.

