# Supplementary figures and images for: Intraspecific variability in the filter mesh size of suspension feeding organisms: the case of invasive Ponto-Caspian corophiids (Crustacea: Amphipoda)
Source: PeerJ. 2021 Apr 19;9:e11245. doi: 10.7717/peerj.11245 (PMC8061577; doi:10.7717/peerj.11245)

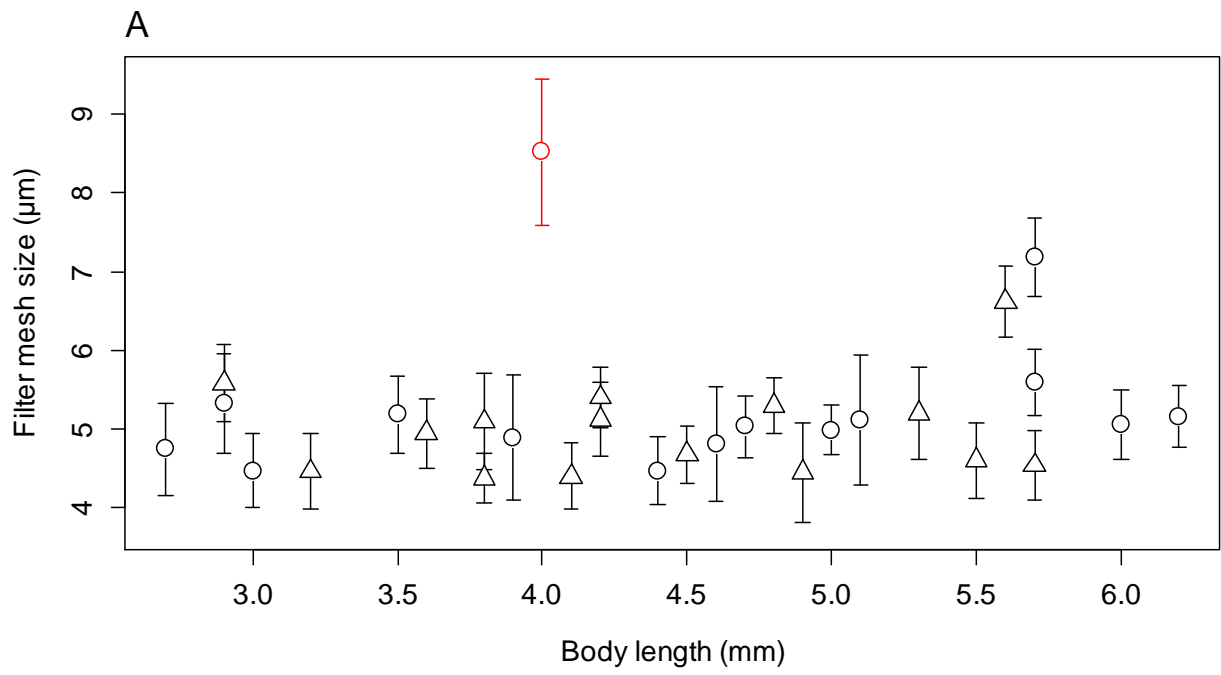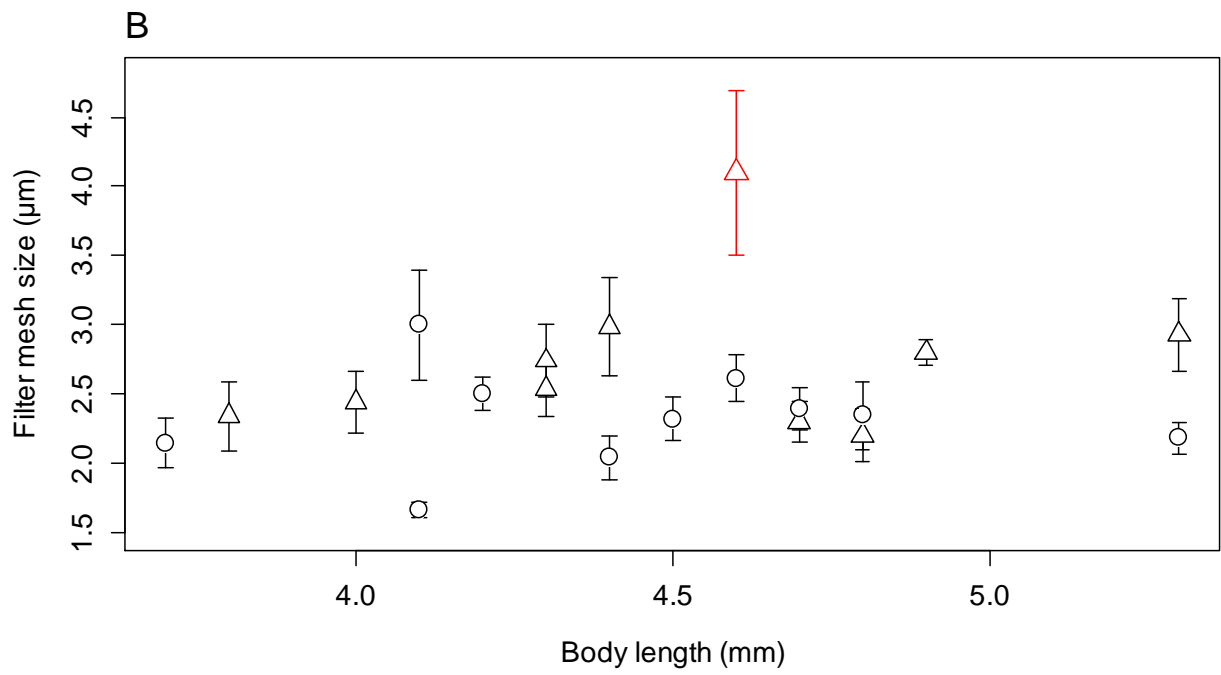

Supplement: Supplemental Information 1 — (A) C. curvispinum in Sample no. 8 (as in Table 1). (B) C. sowinskyi in Sample no. 33. Circles: females, triangles: males. The individuals considered outliers are marked red. The FMS of the individual in (A) is outstanding only in the sample. This sampling site was under the influence of a small tributary; the presence of the outlier might perhaps be a result of drift from a population under different selection pressures. The individual in (B) is a global outlier in the studied material of C. sowinskyi. Its unusually large FMS might potentially be a result of developmental abnormality. [file peerj-09-11245-s001.pdf]

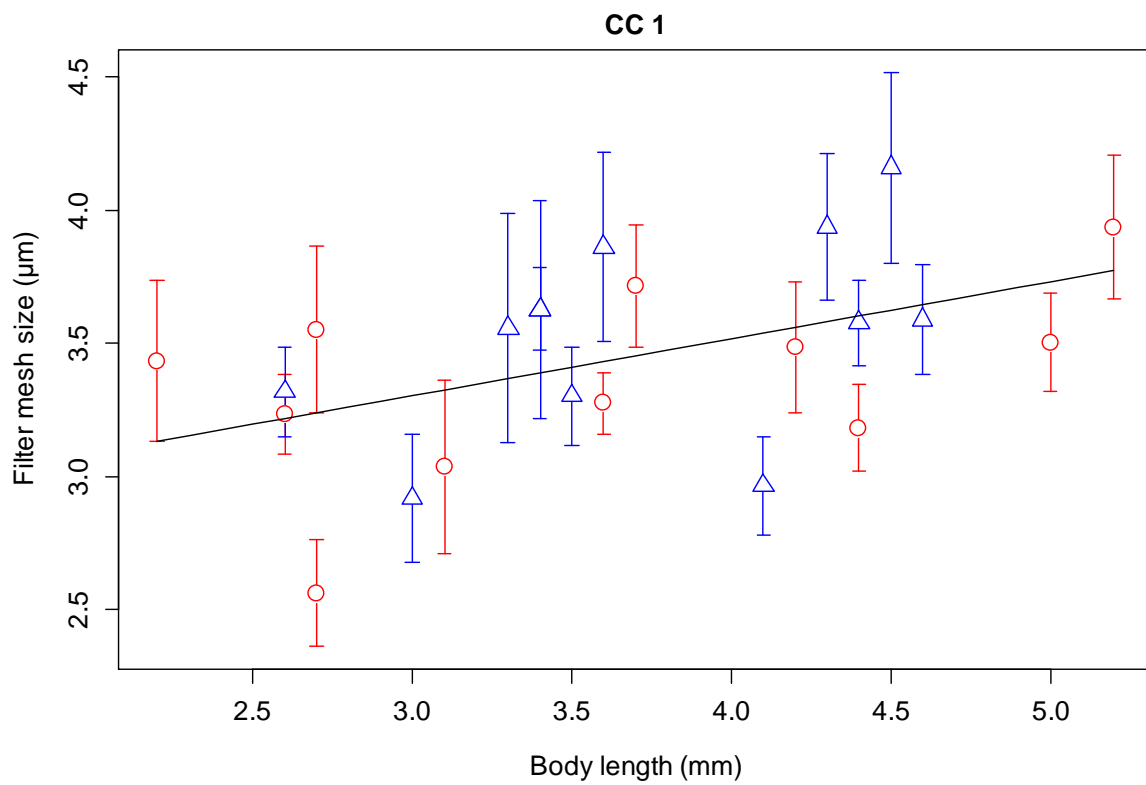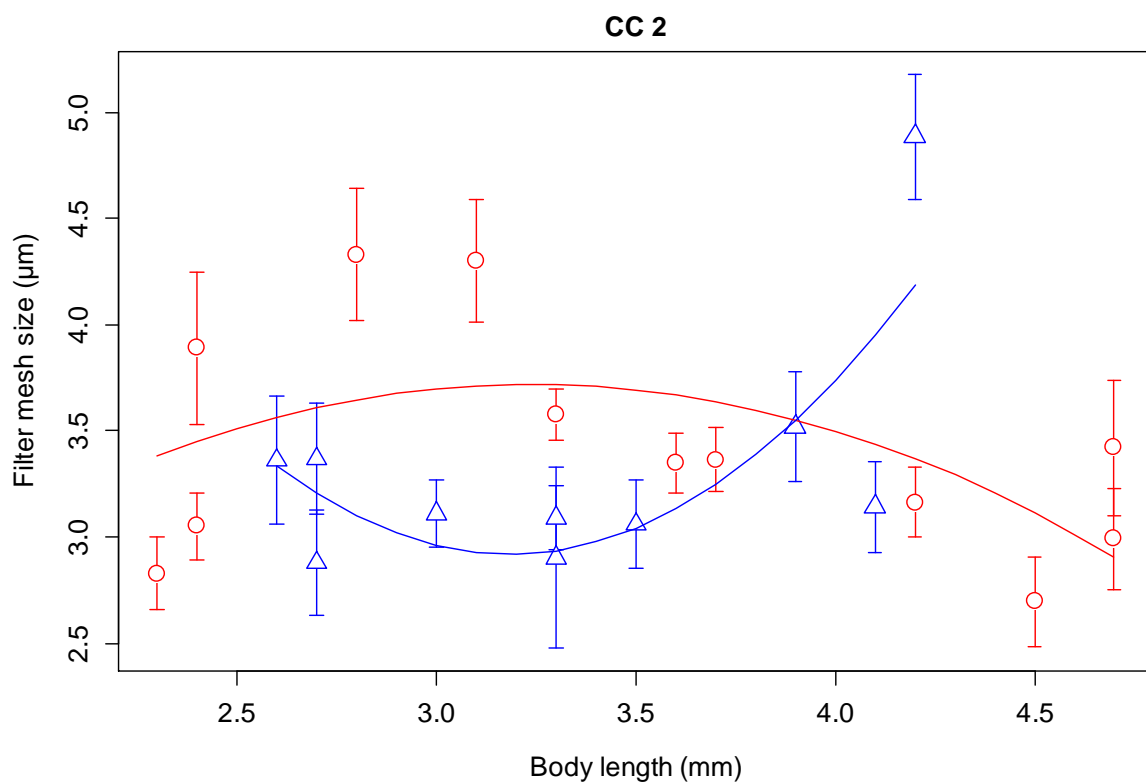

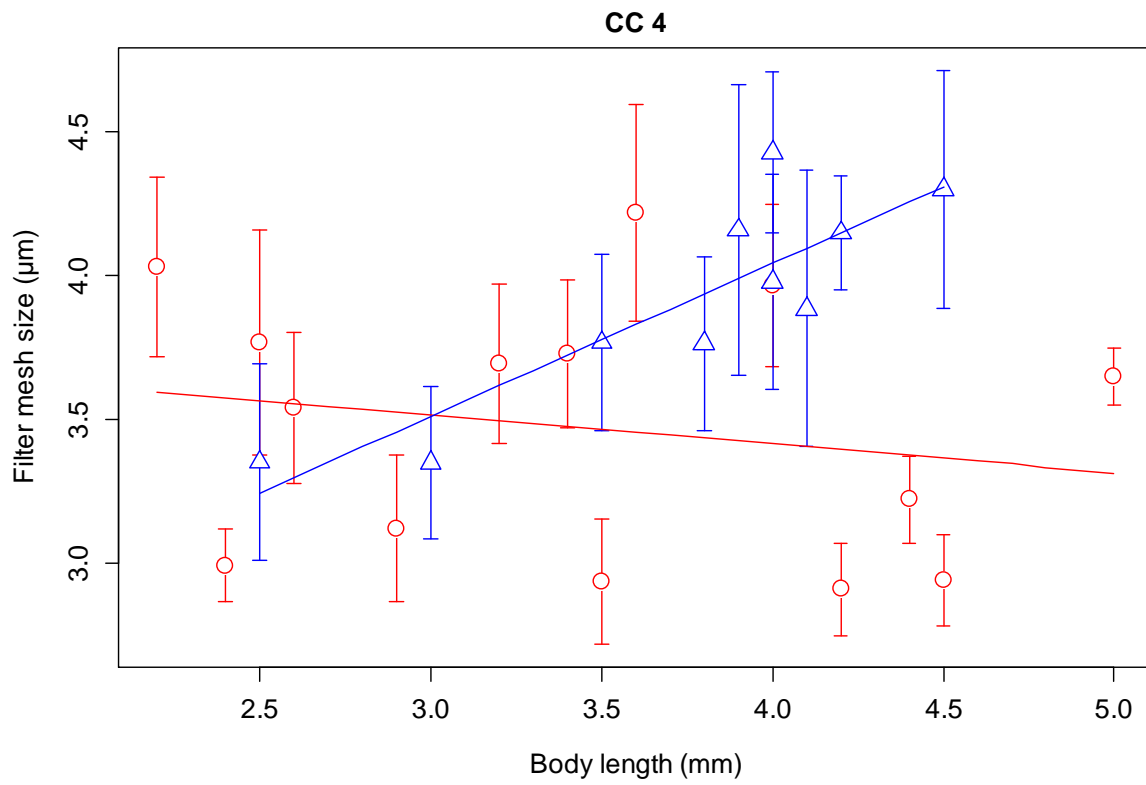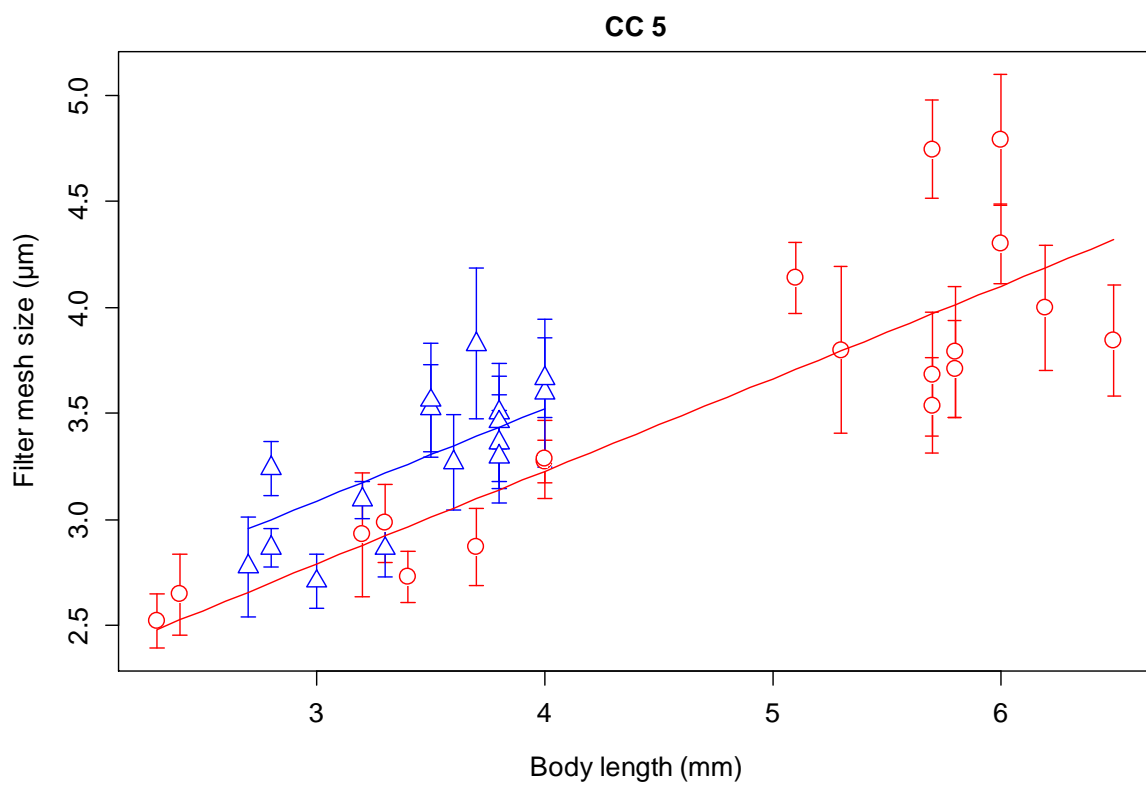

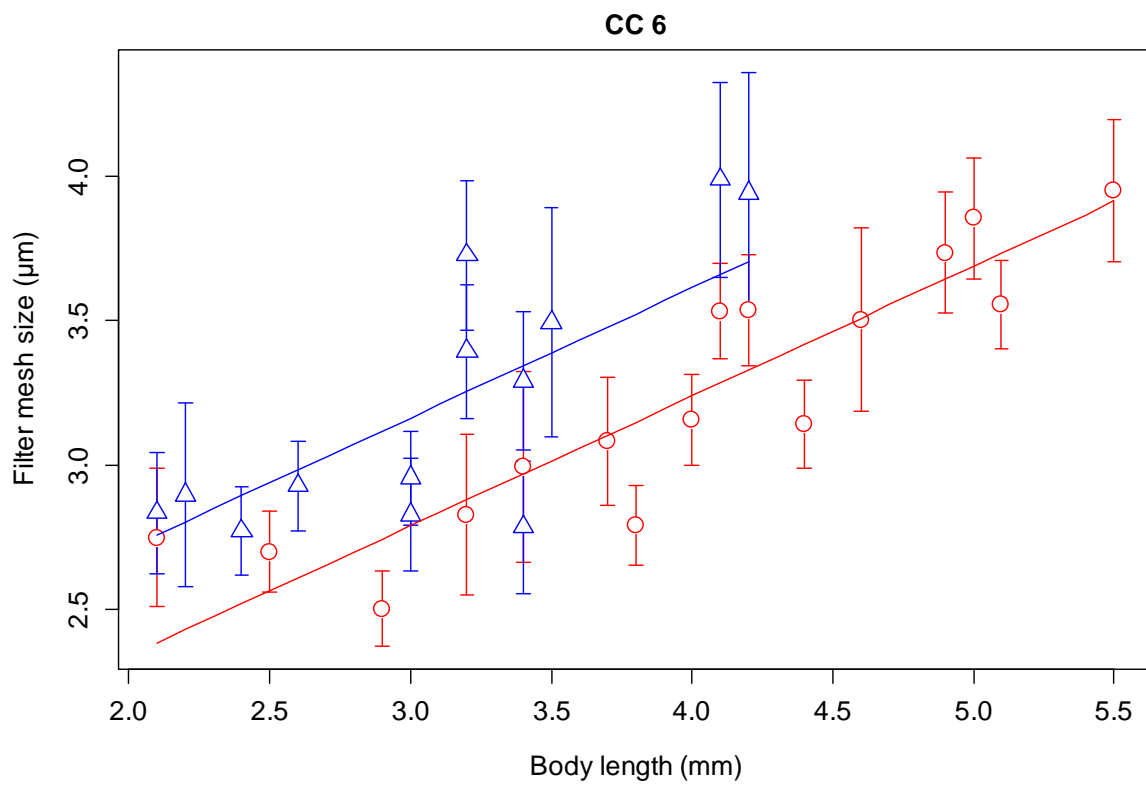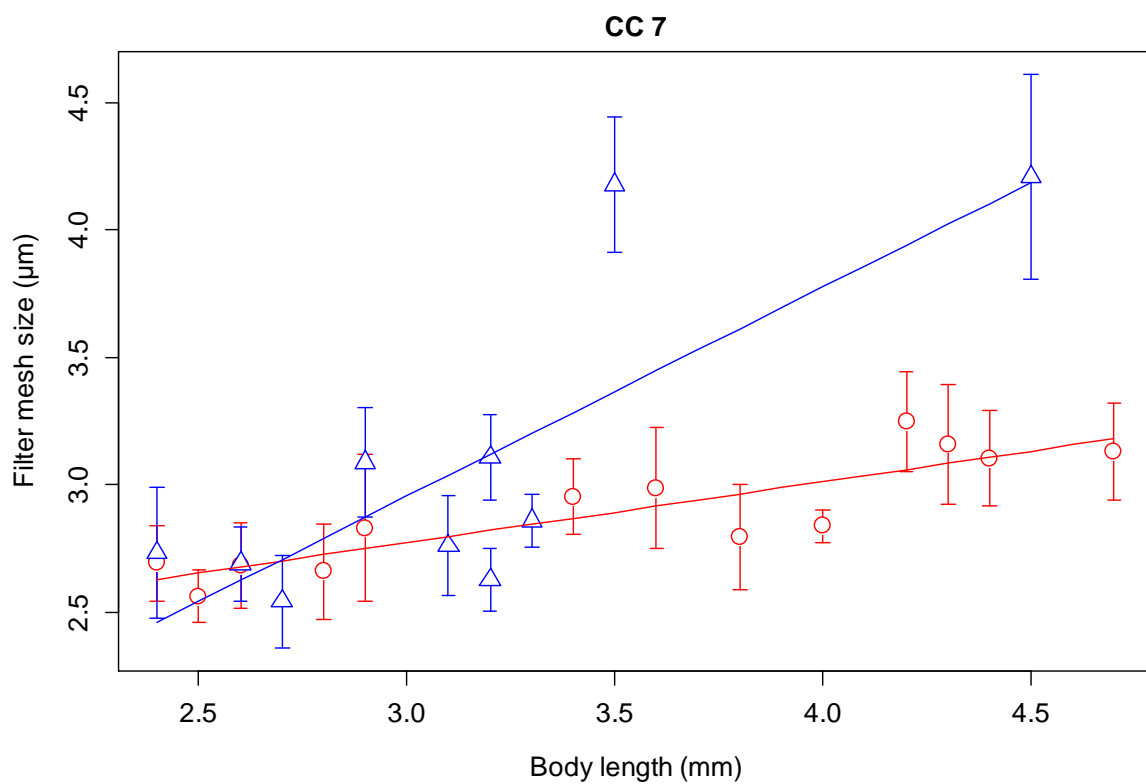

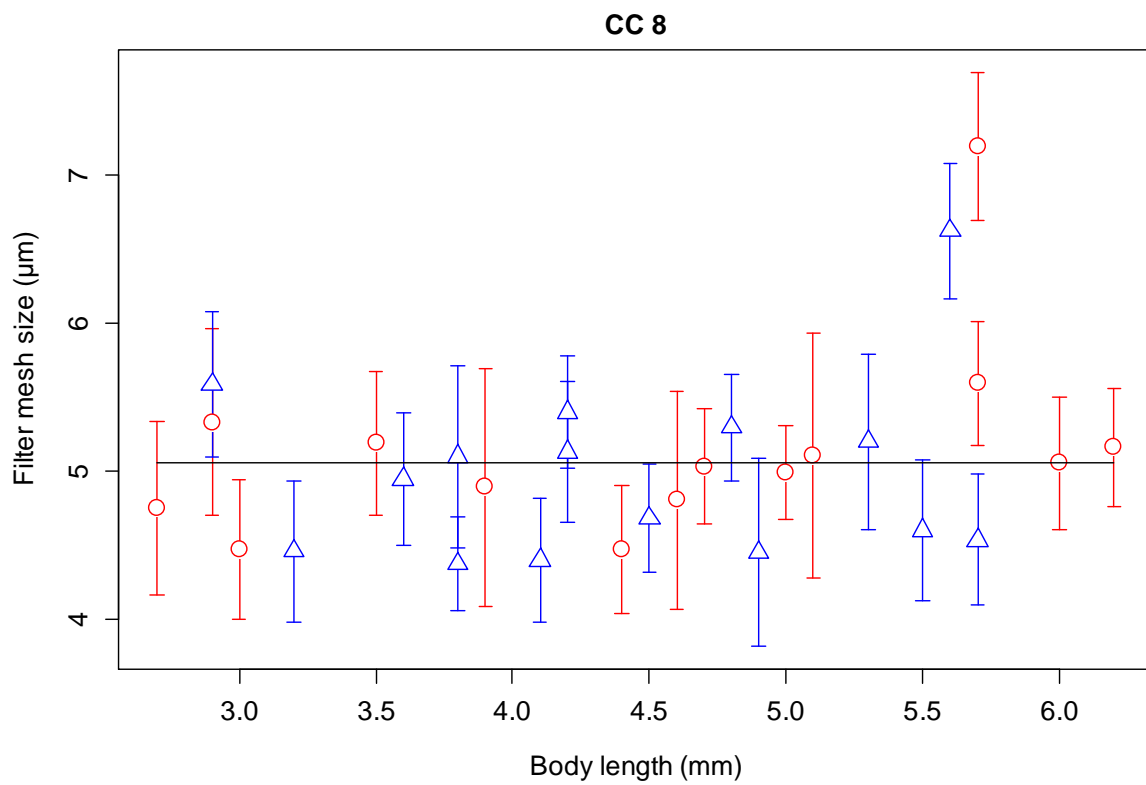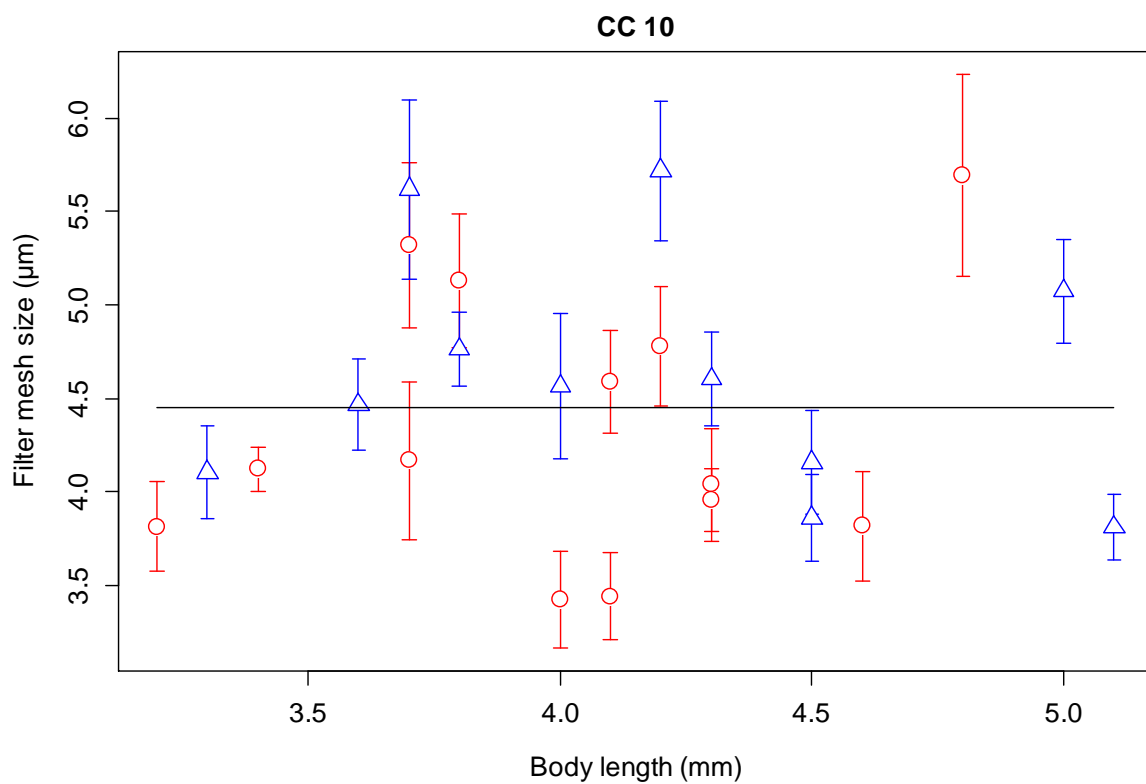

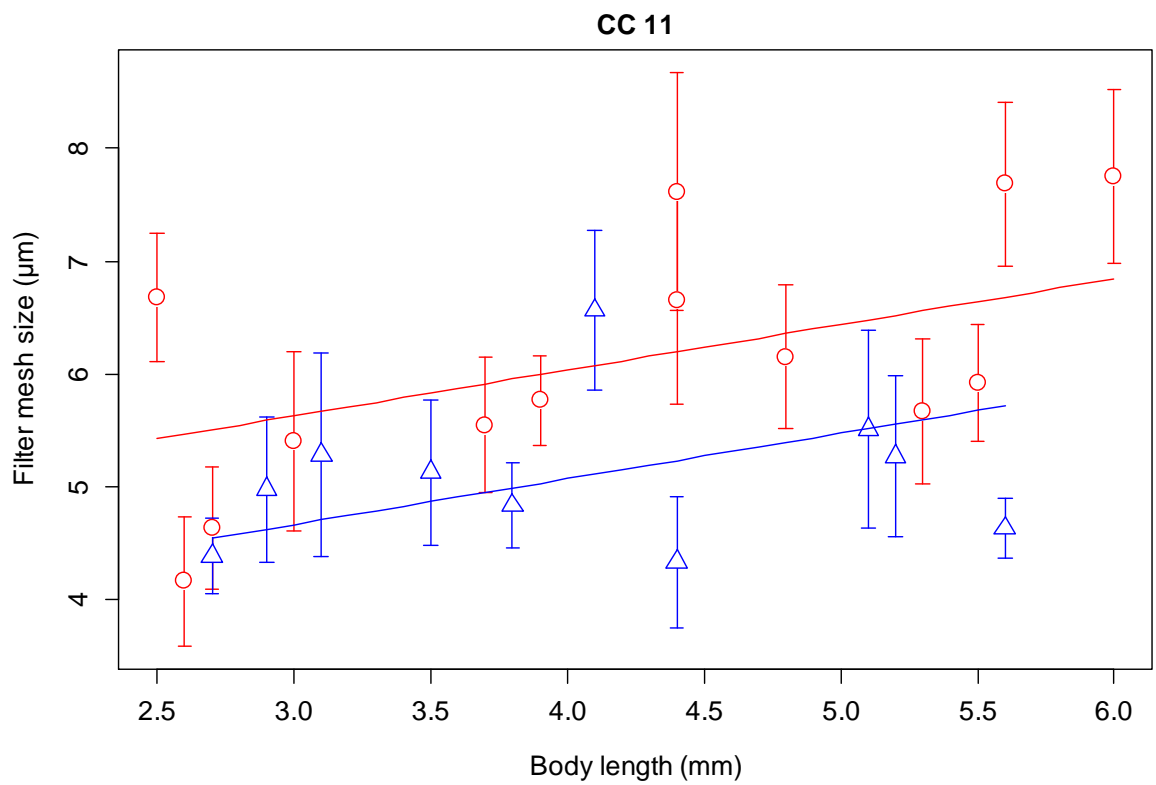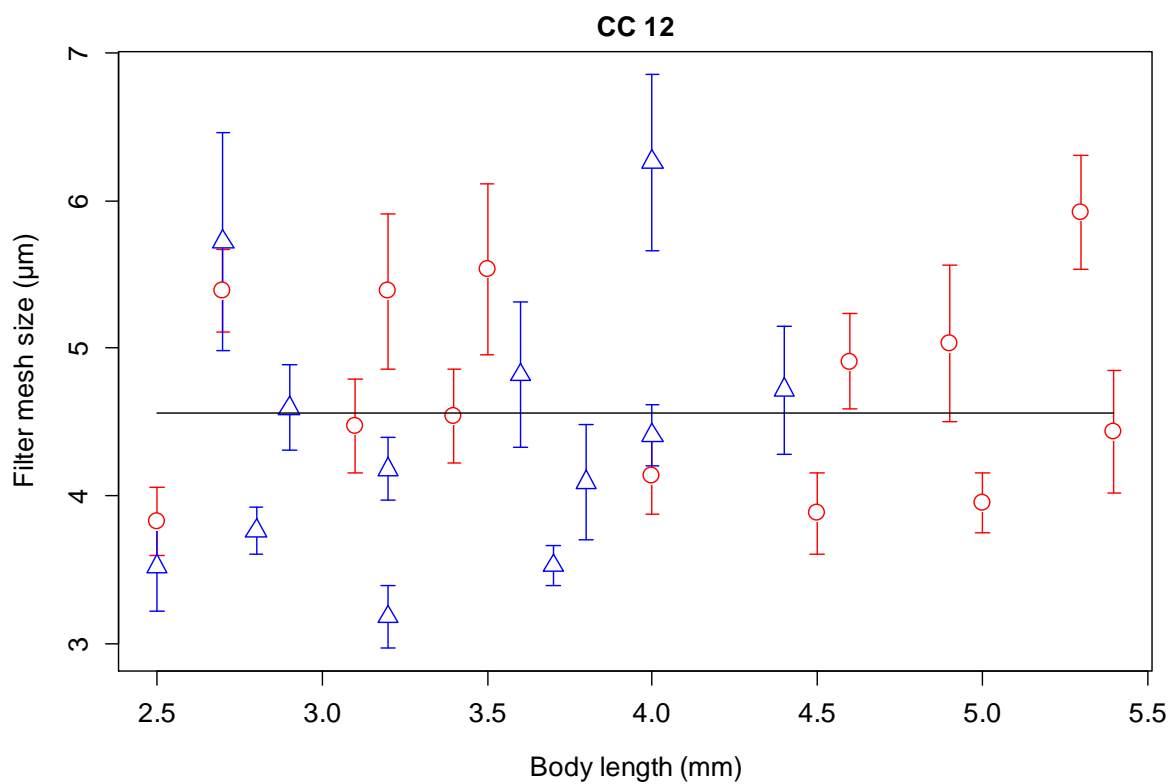

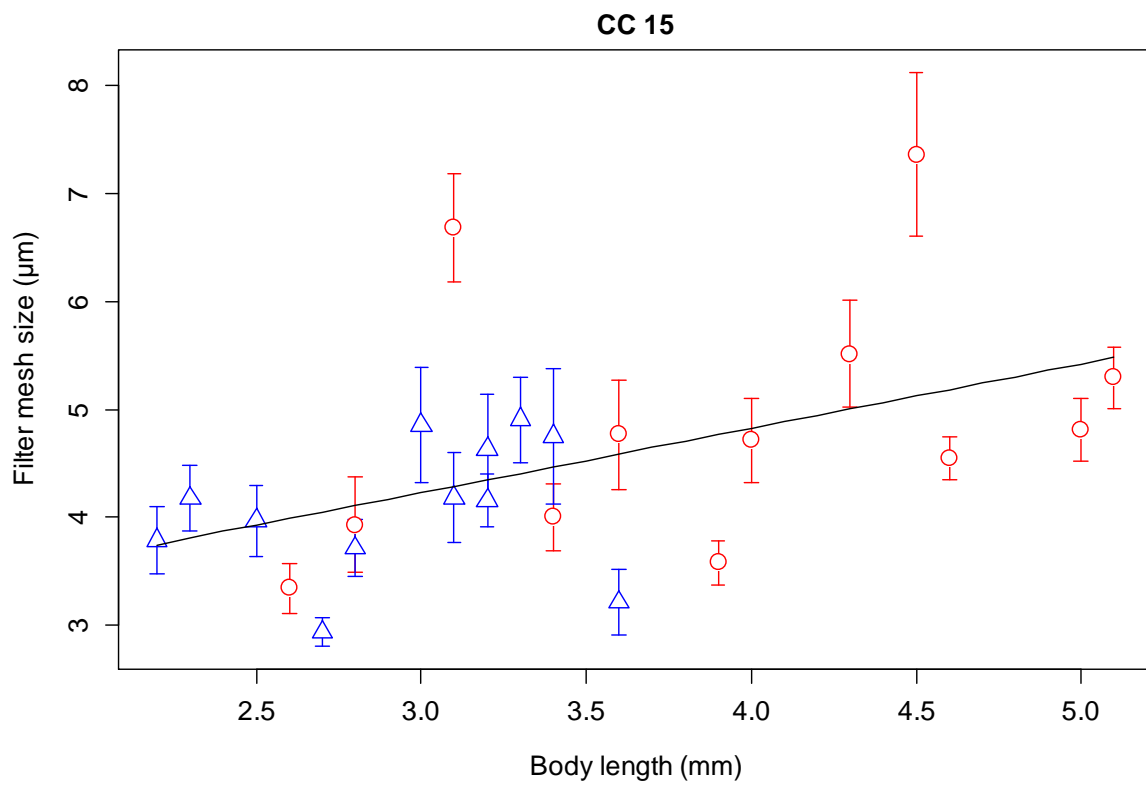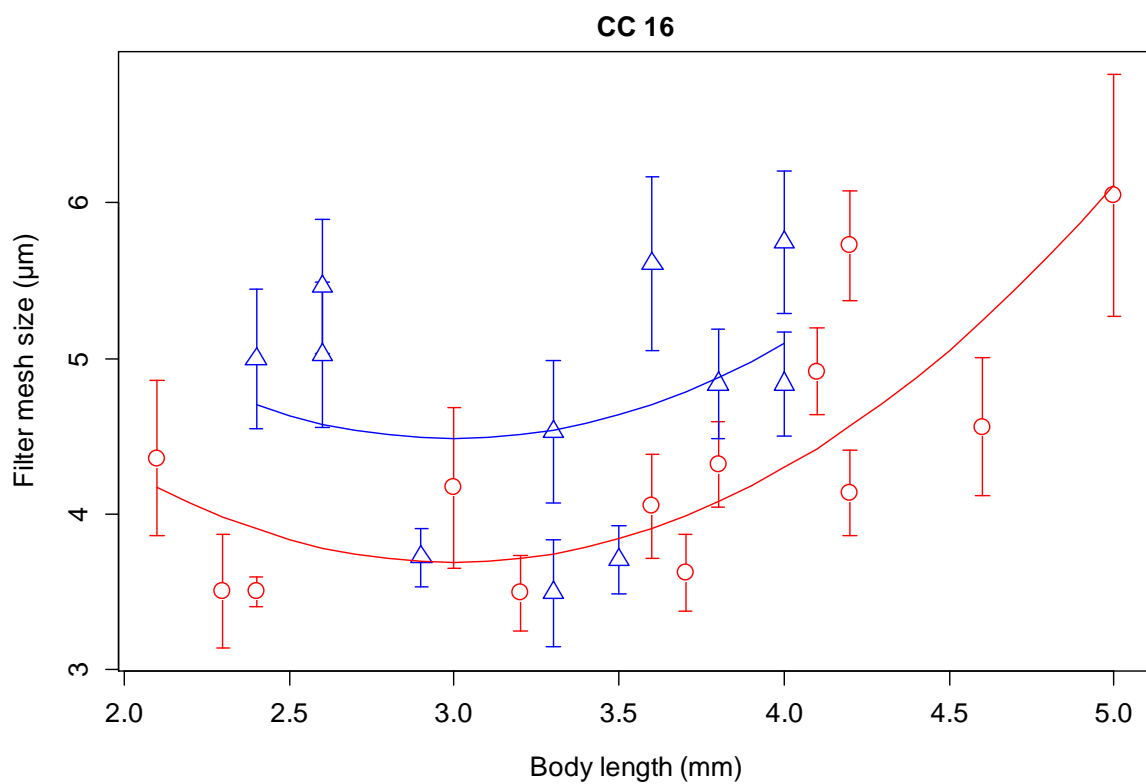

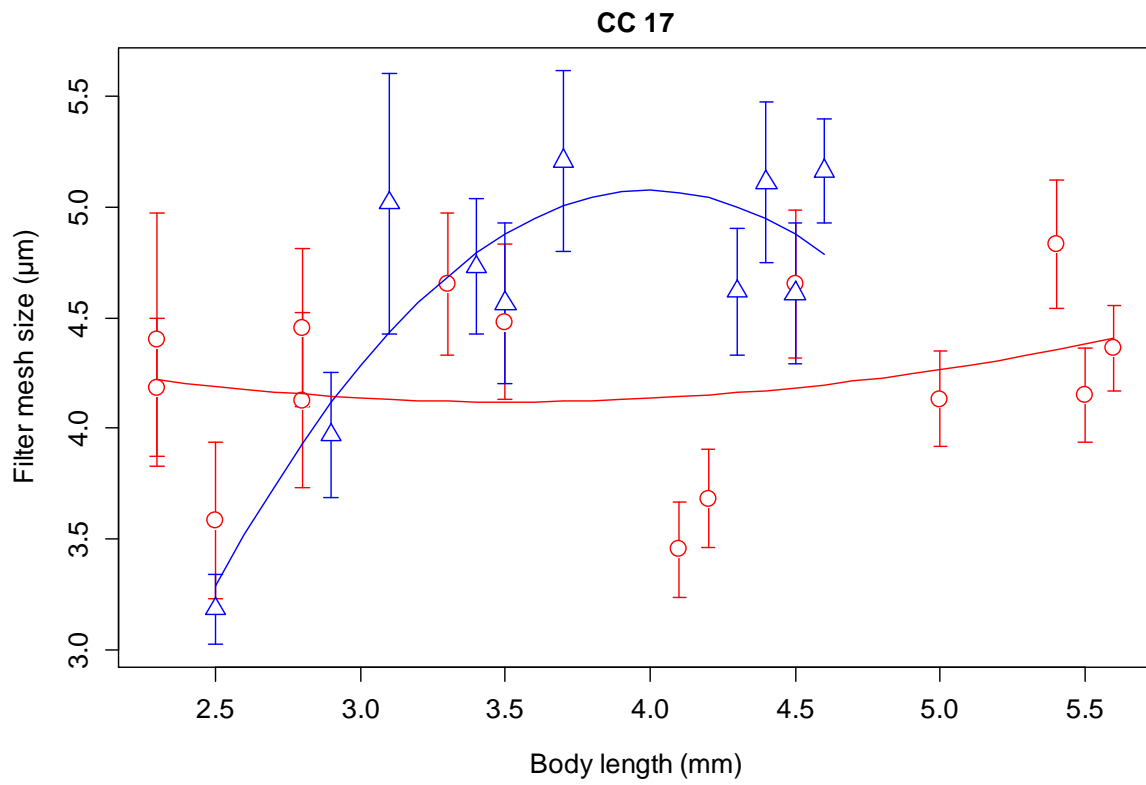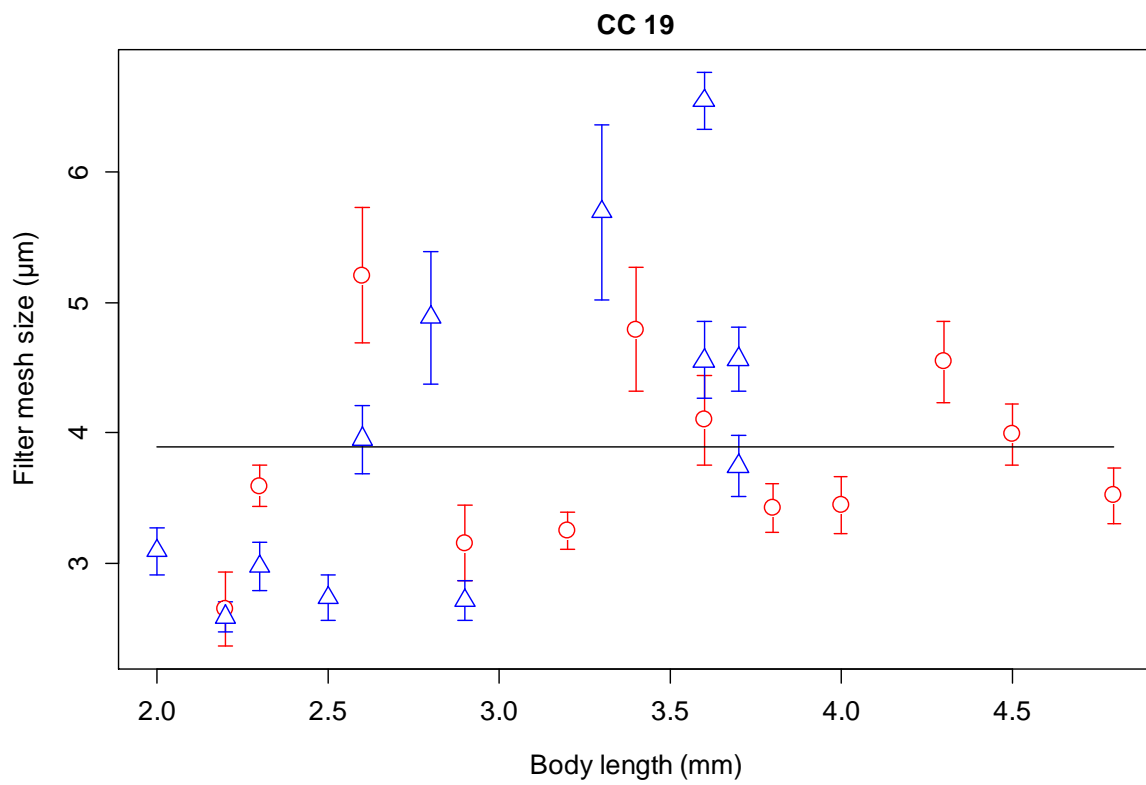

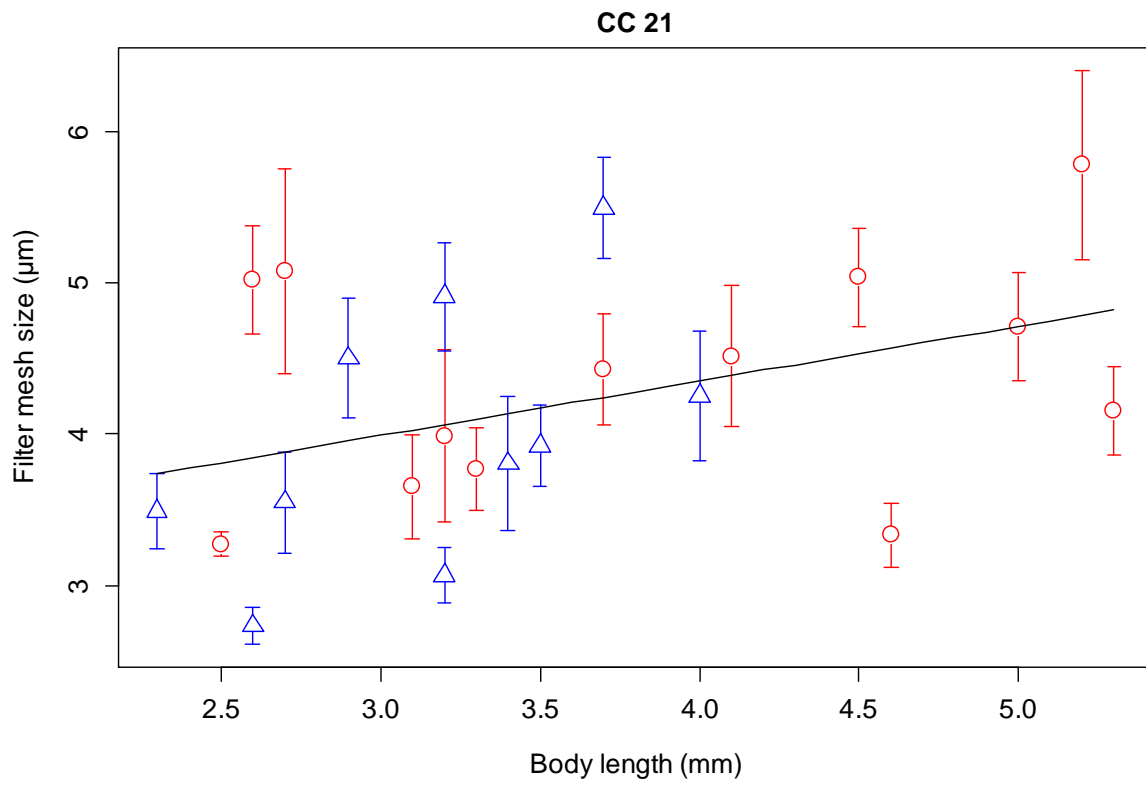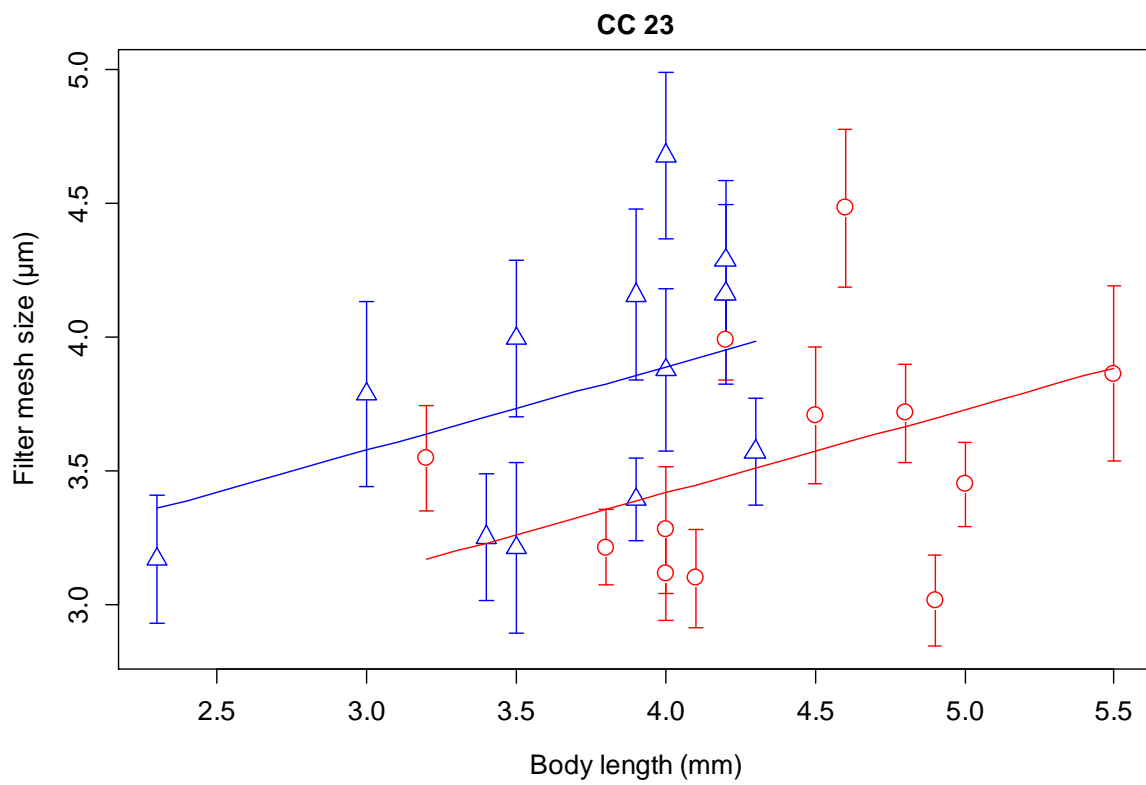

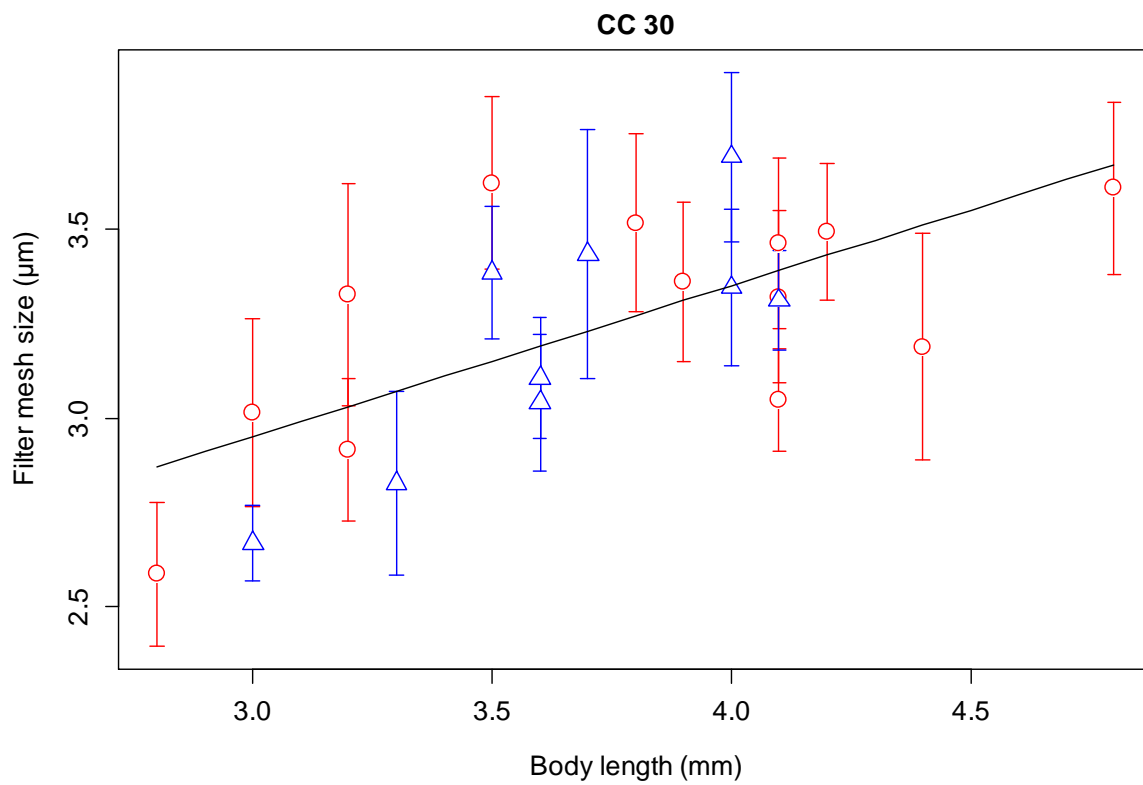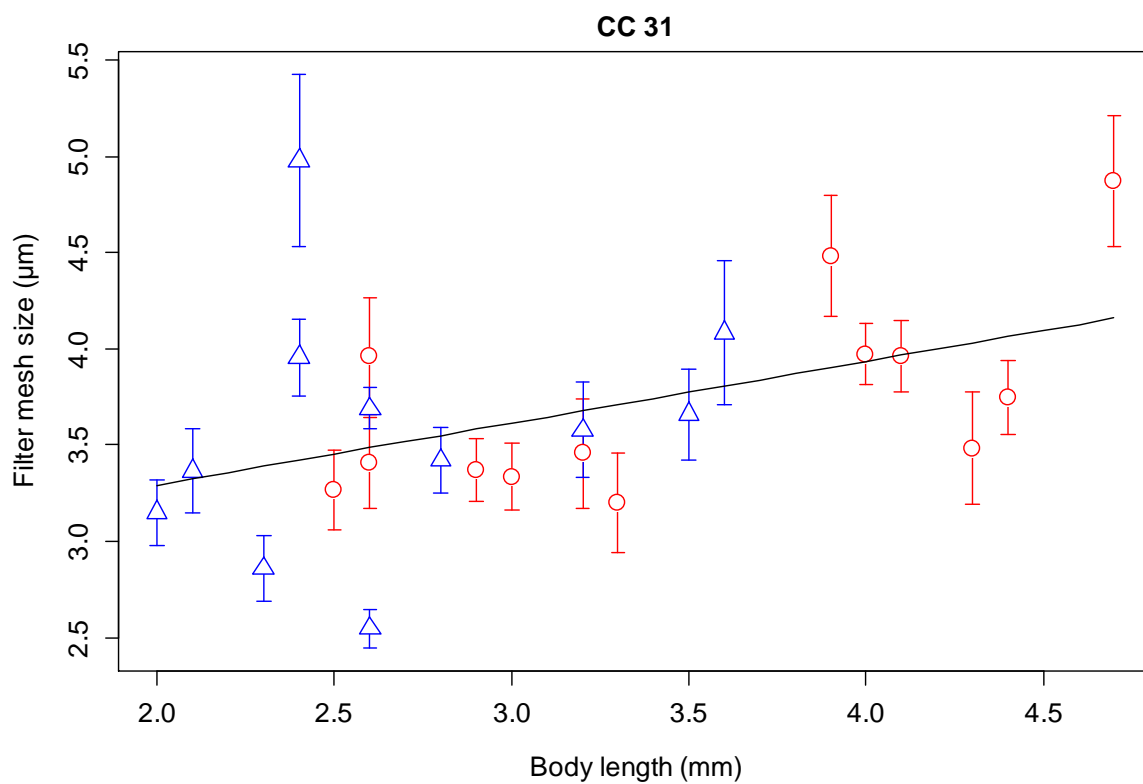

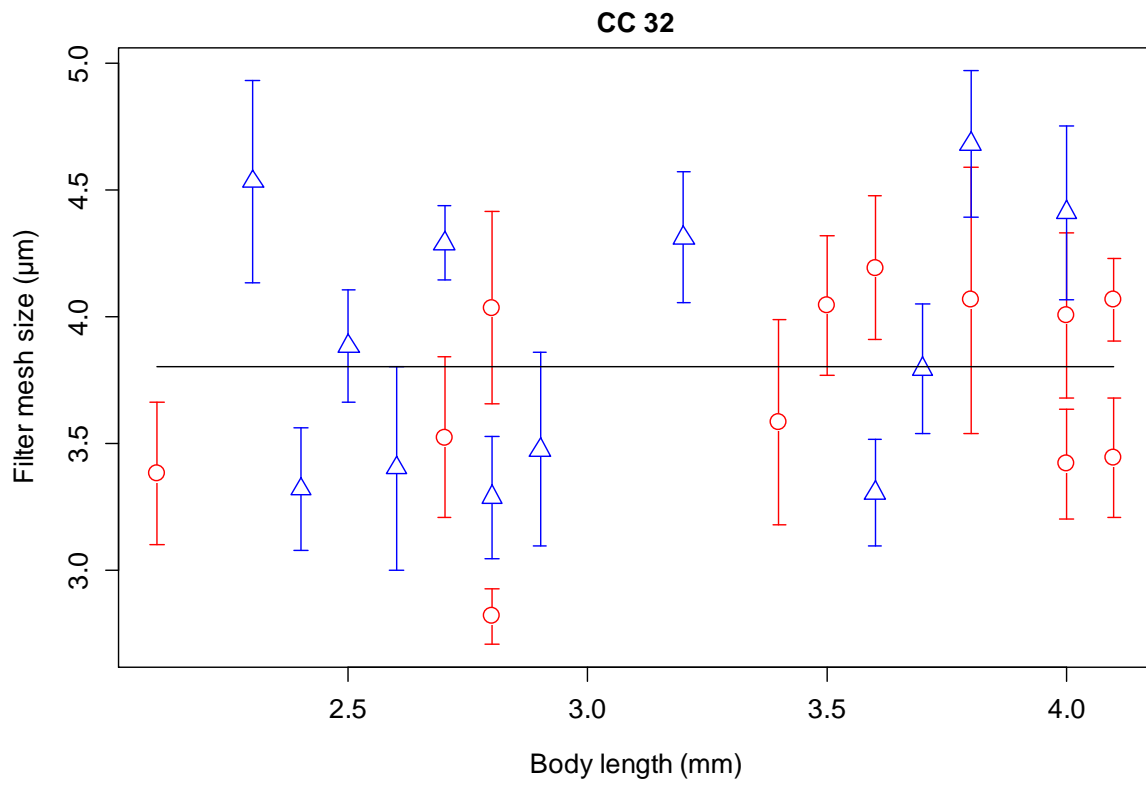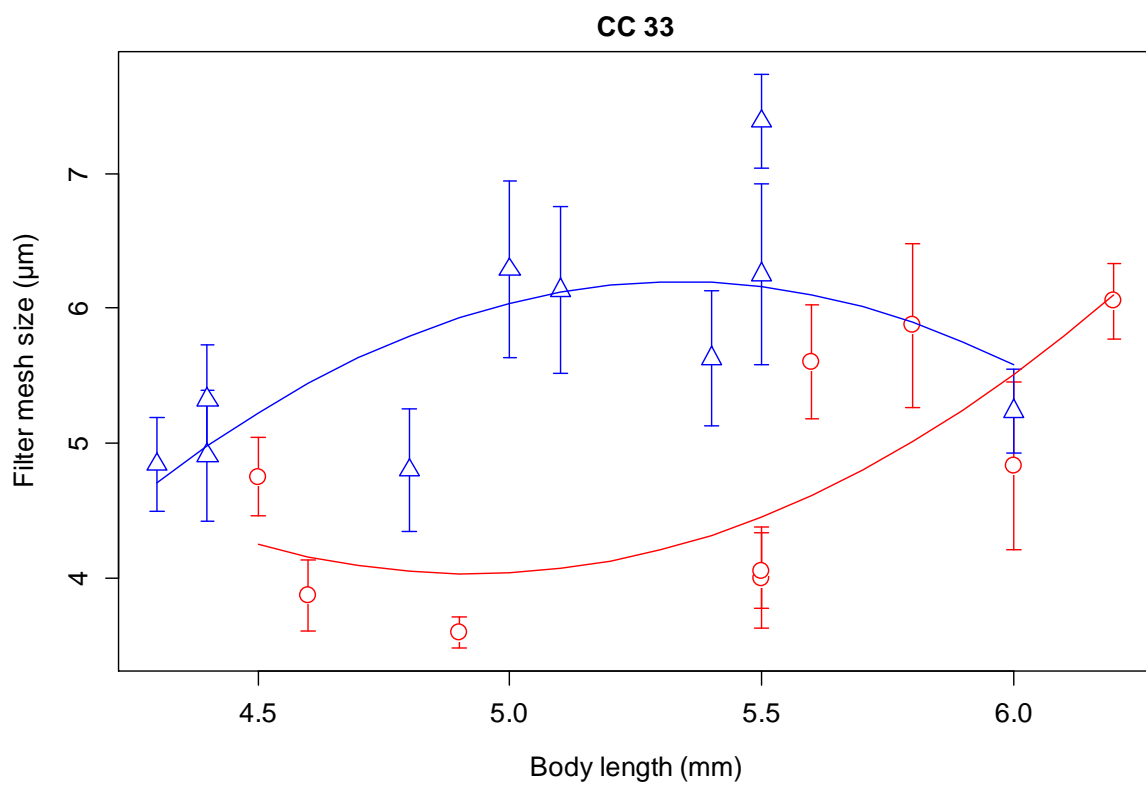

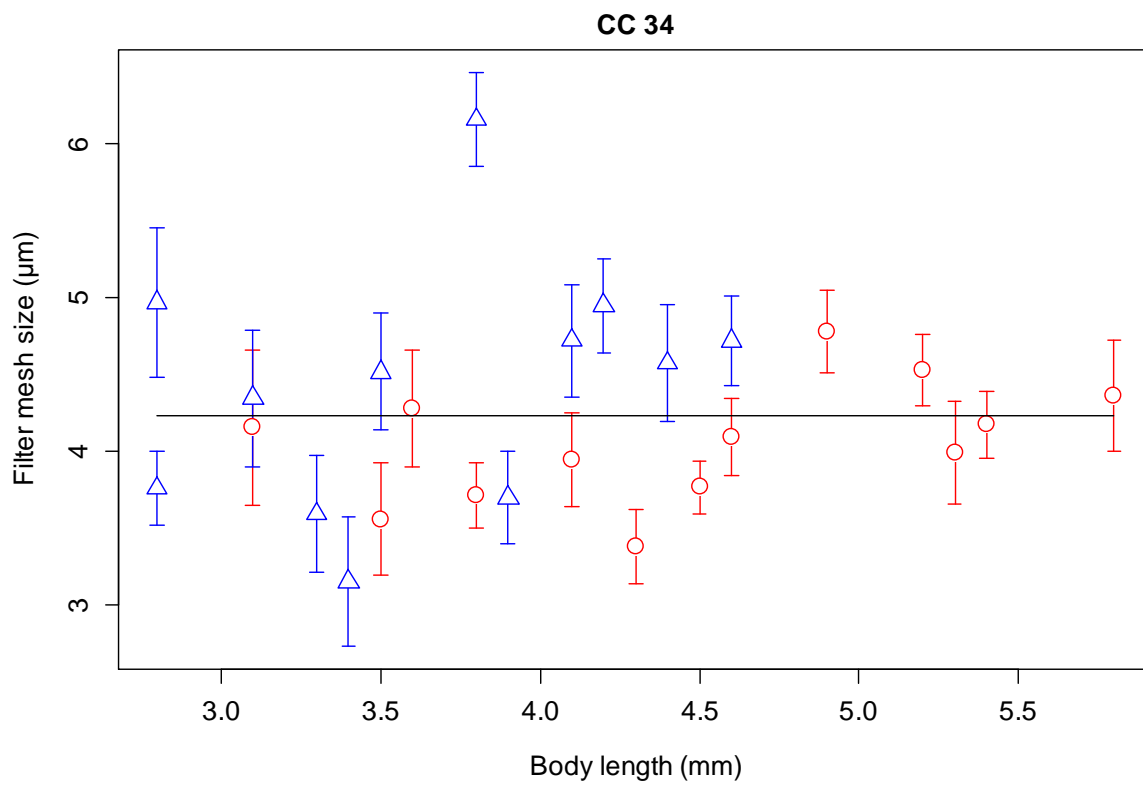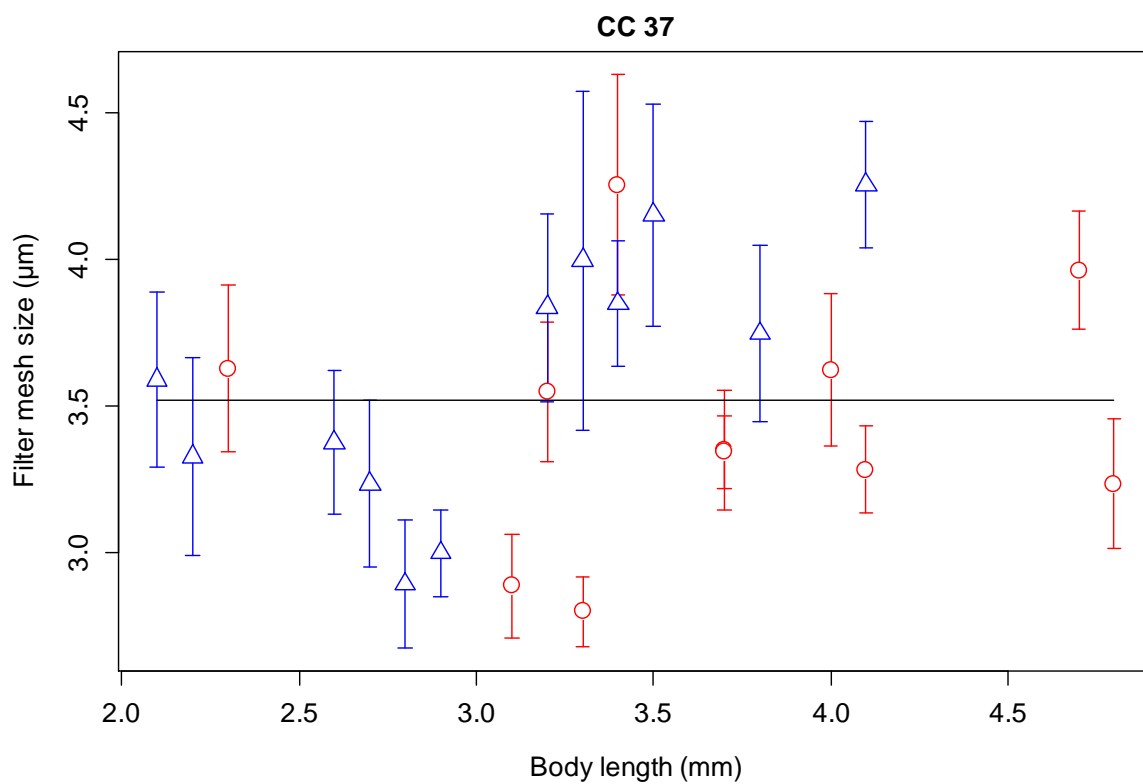

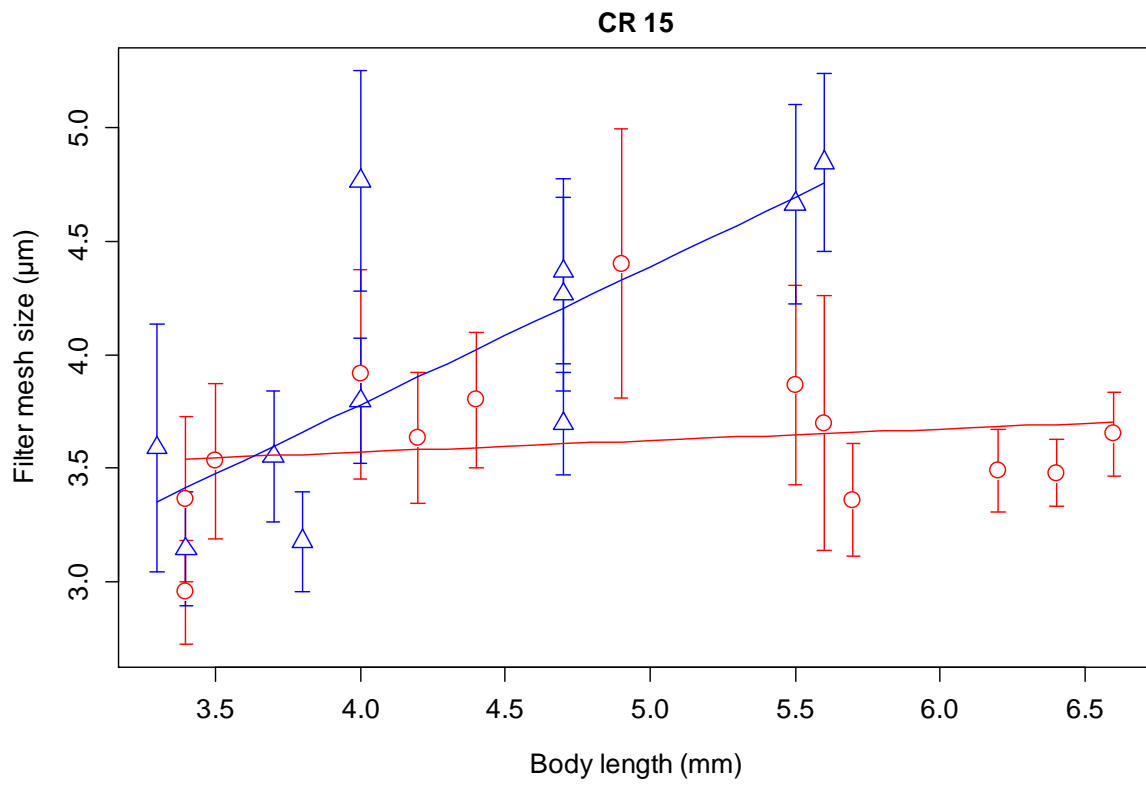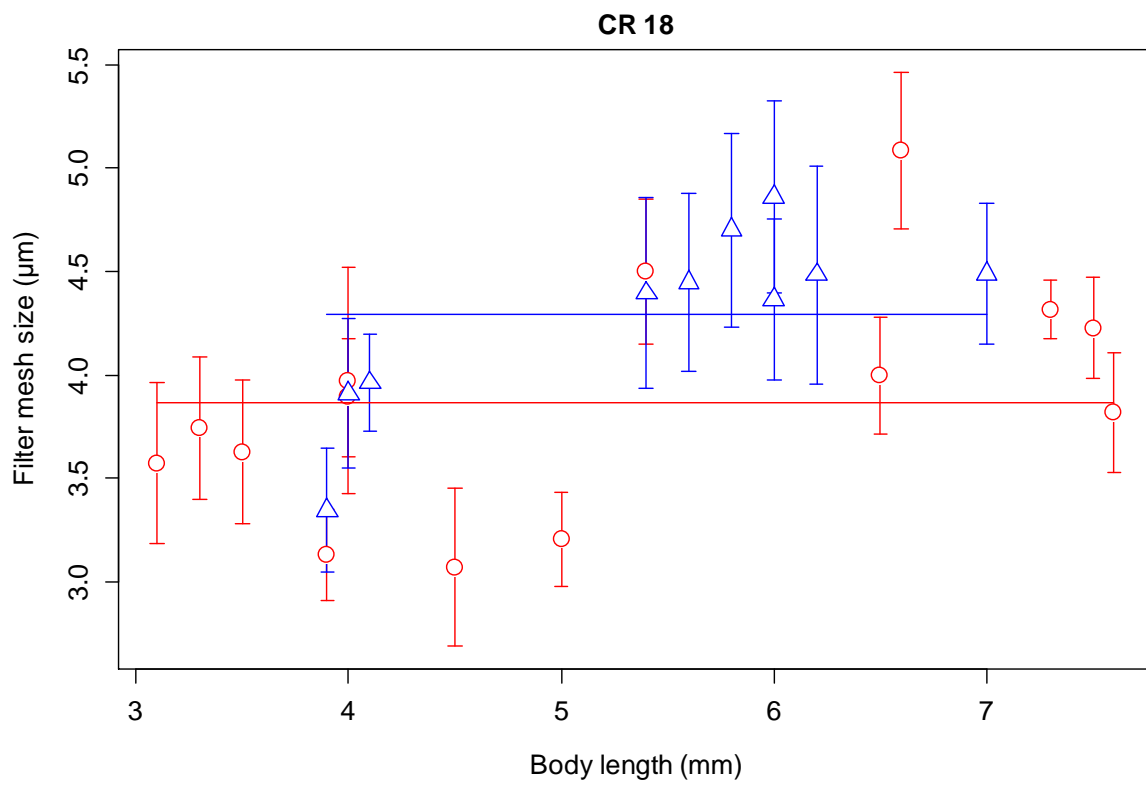

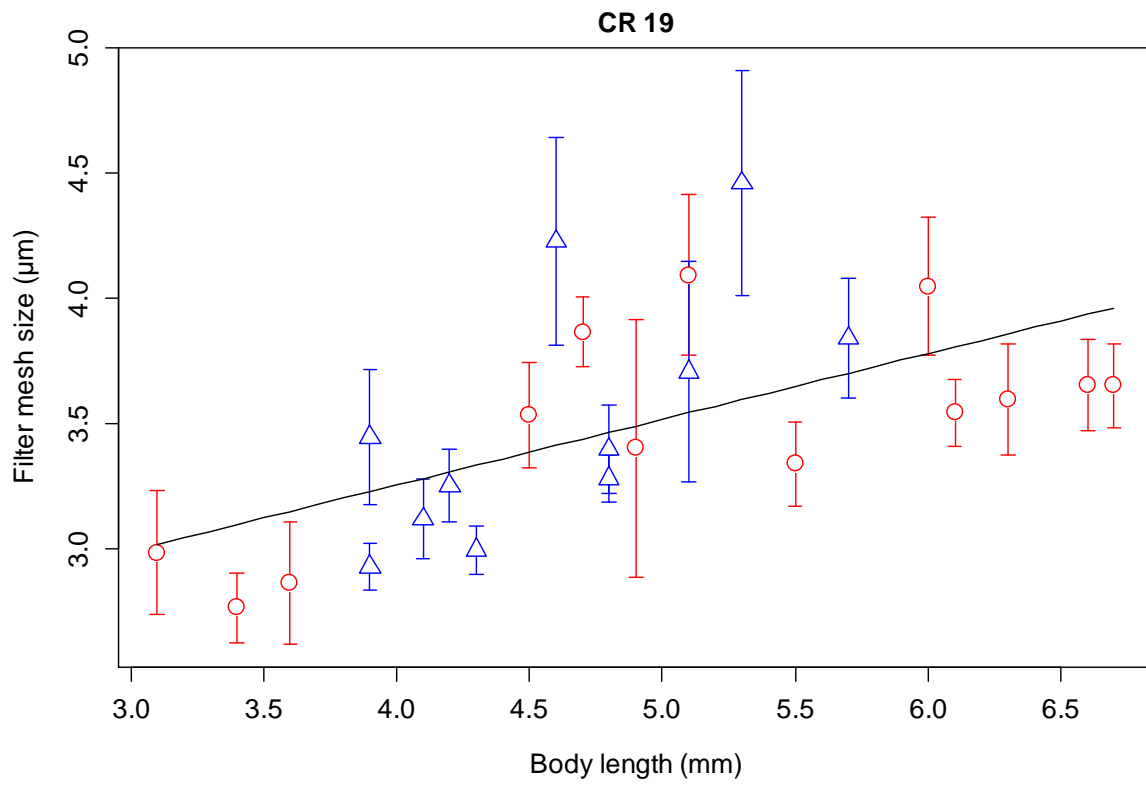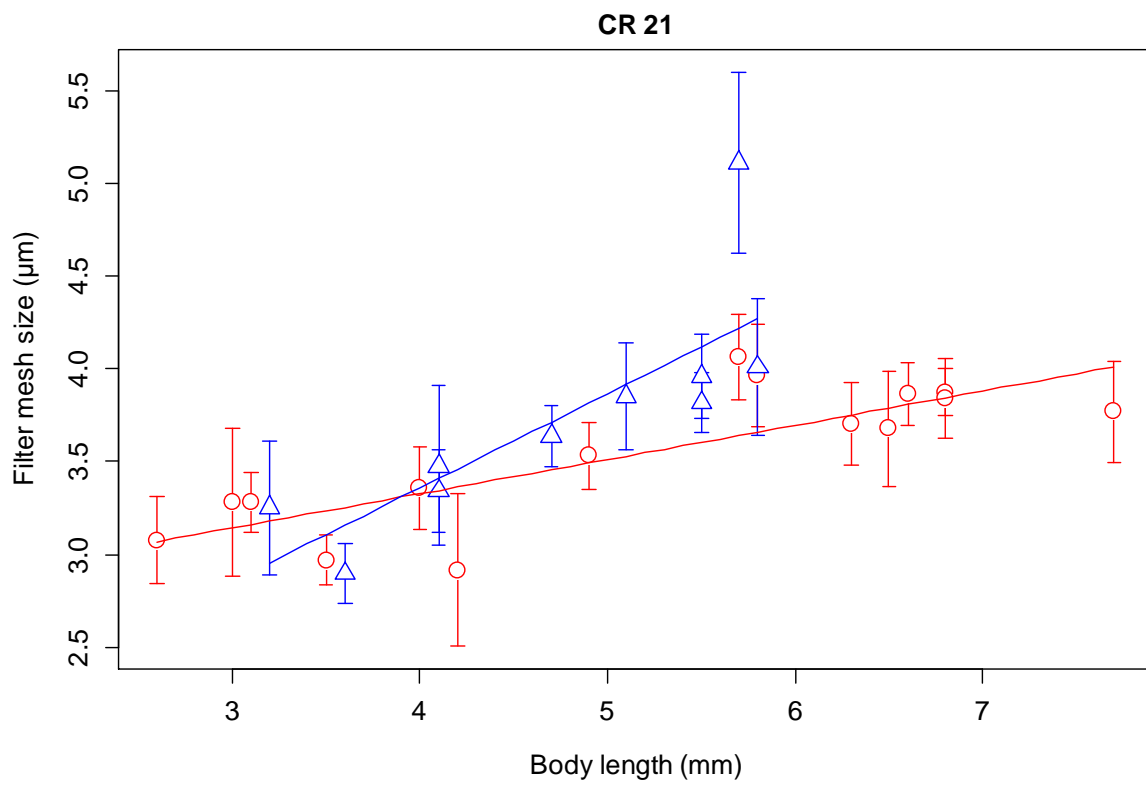

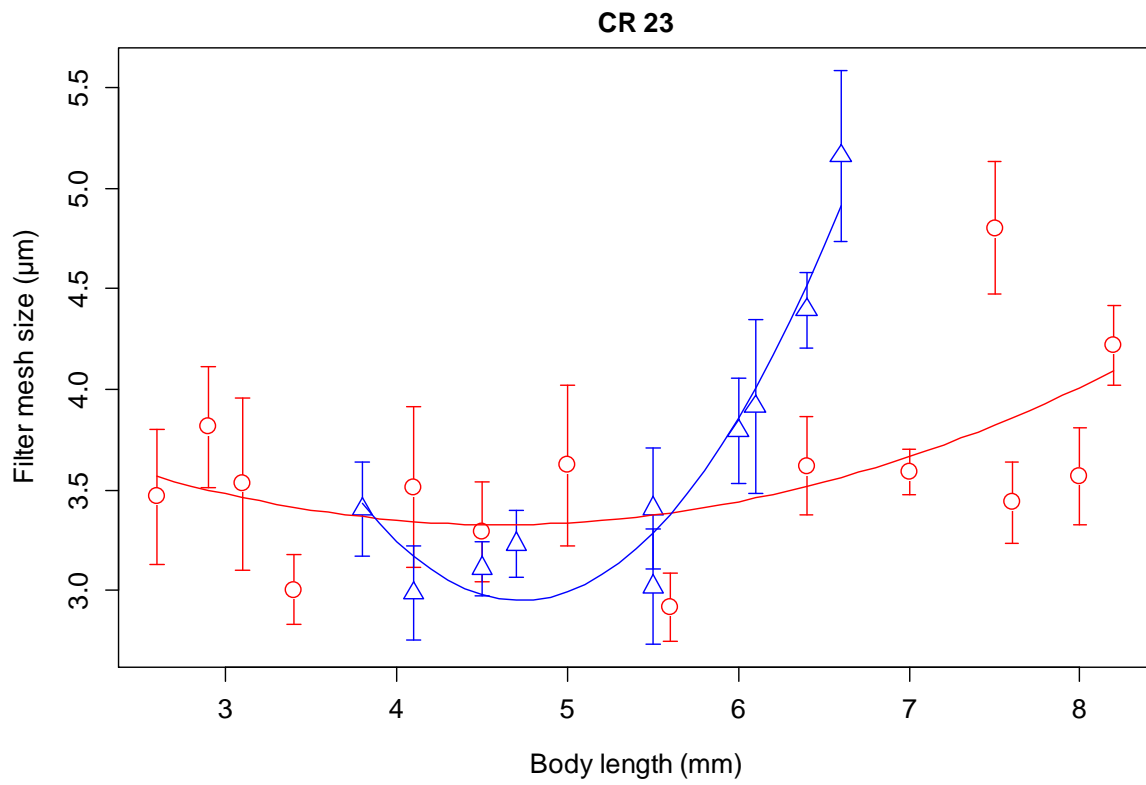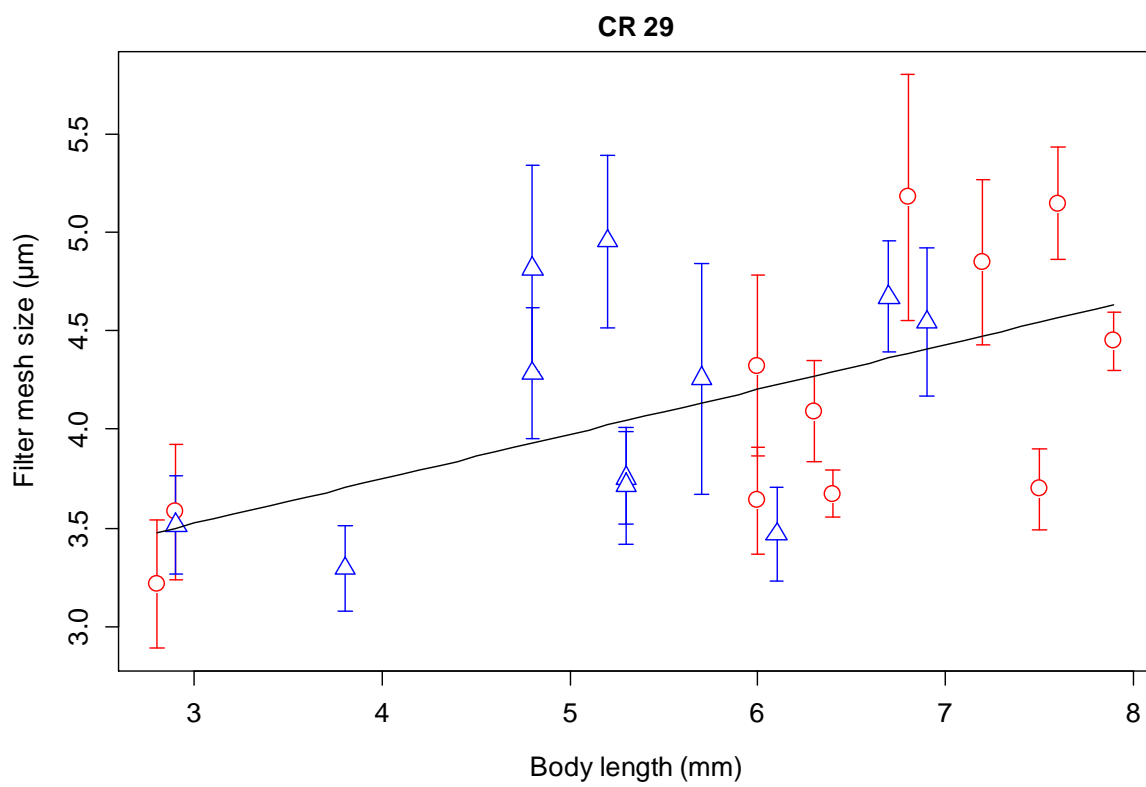

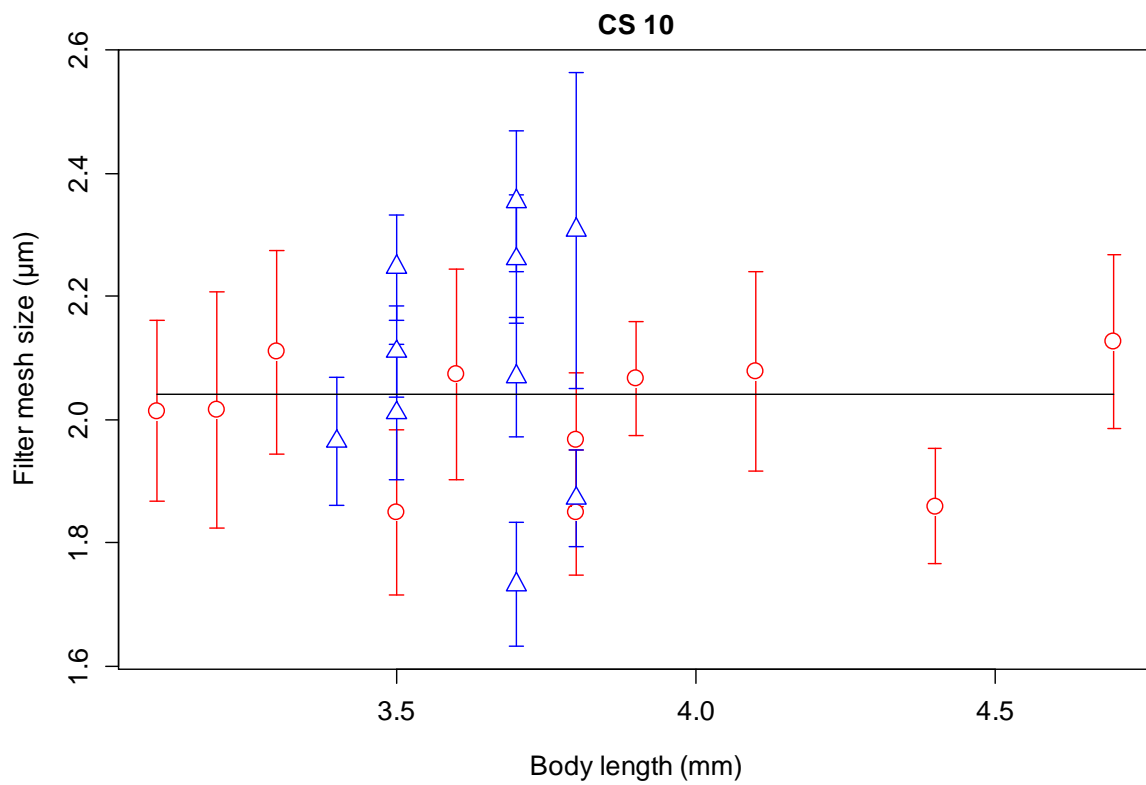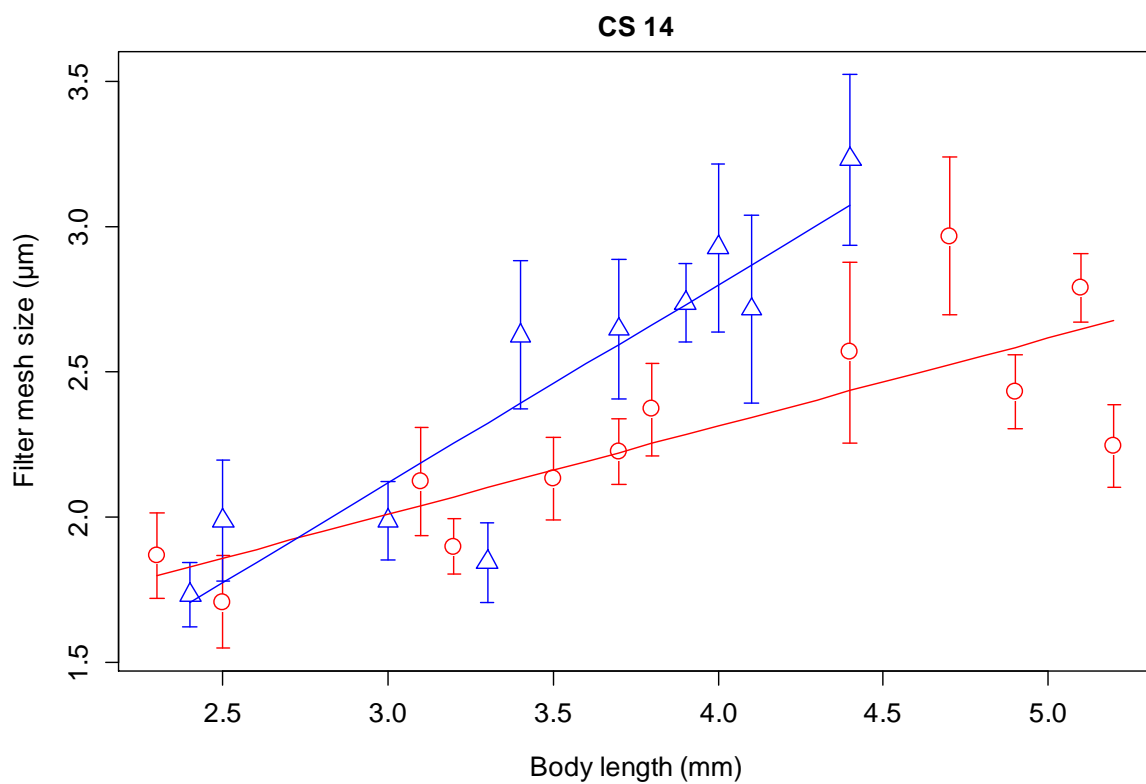

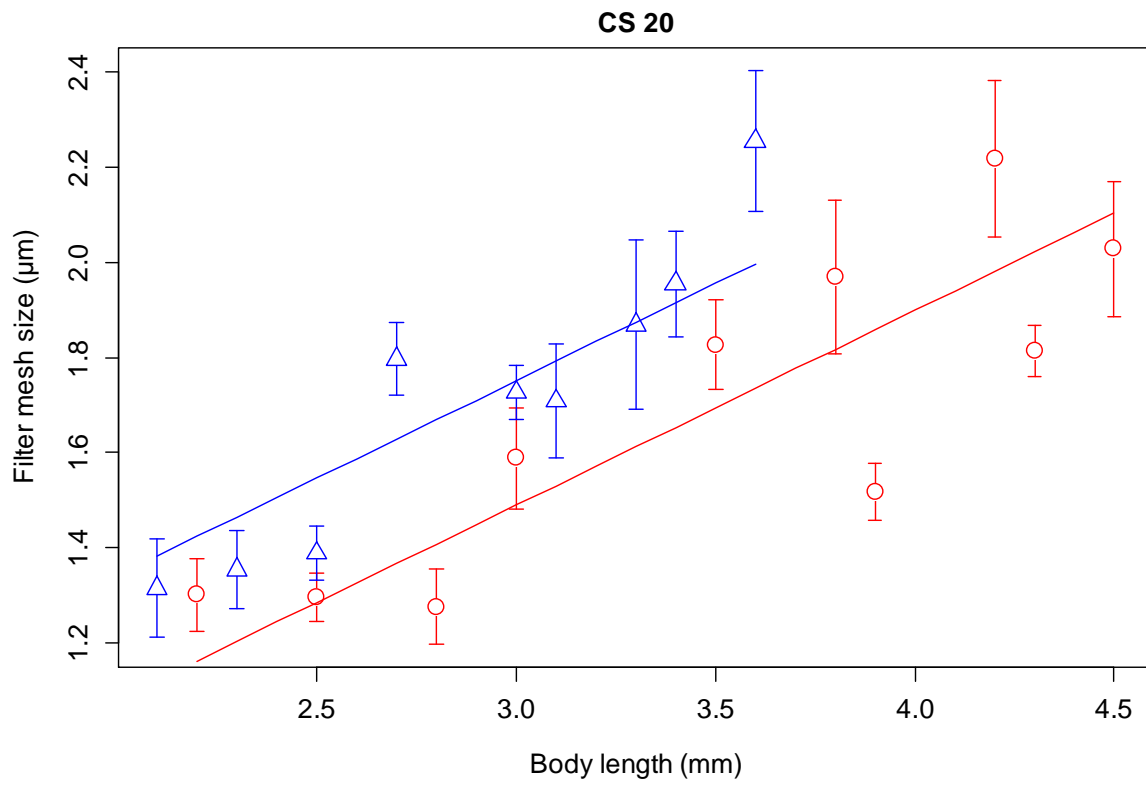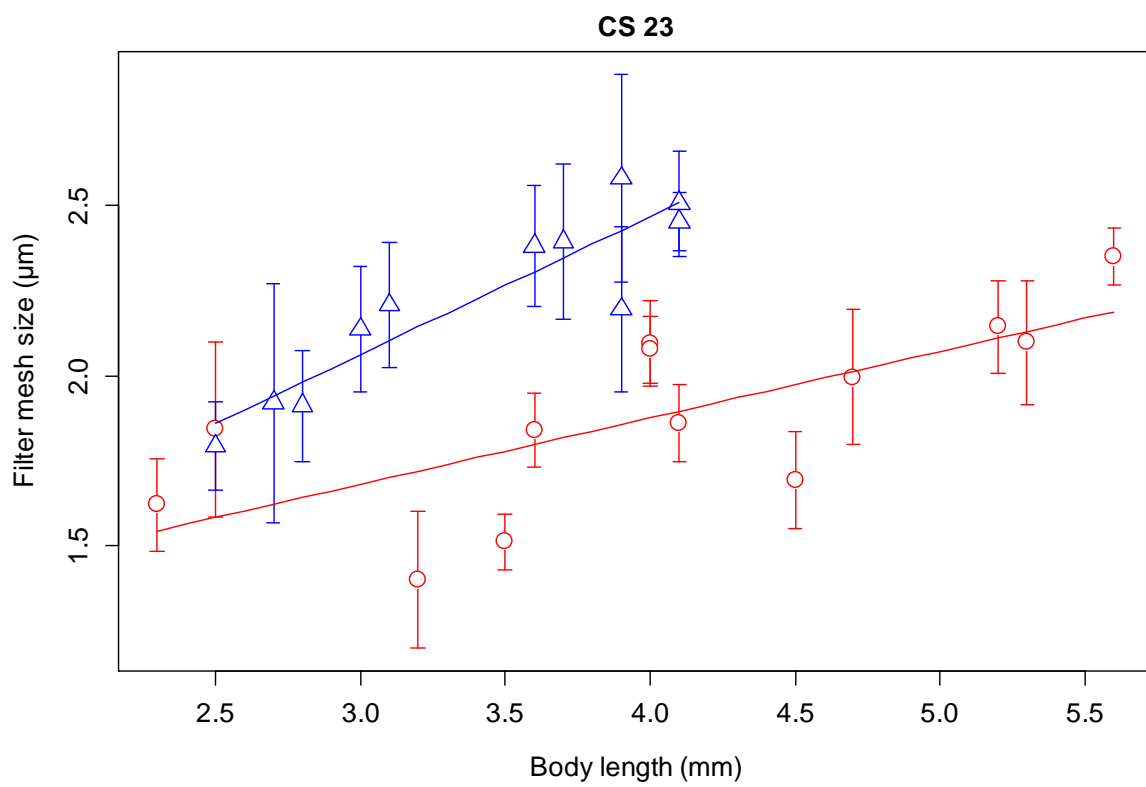

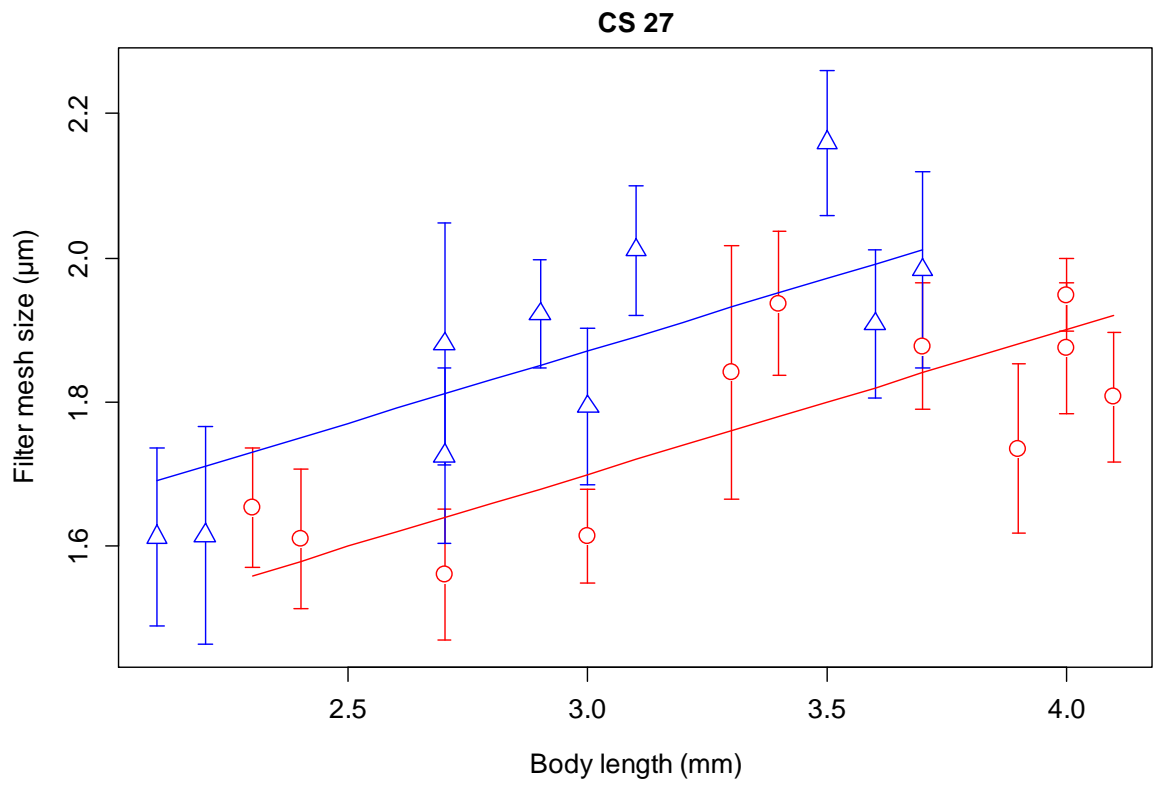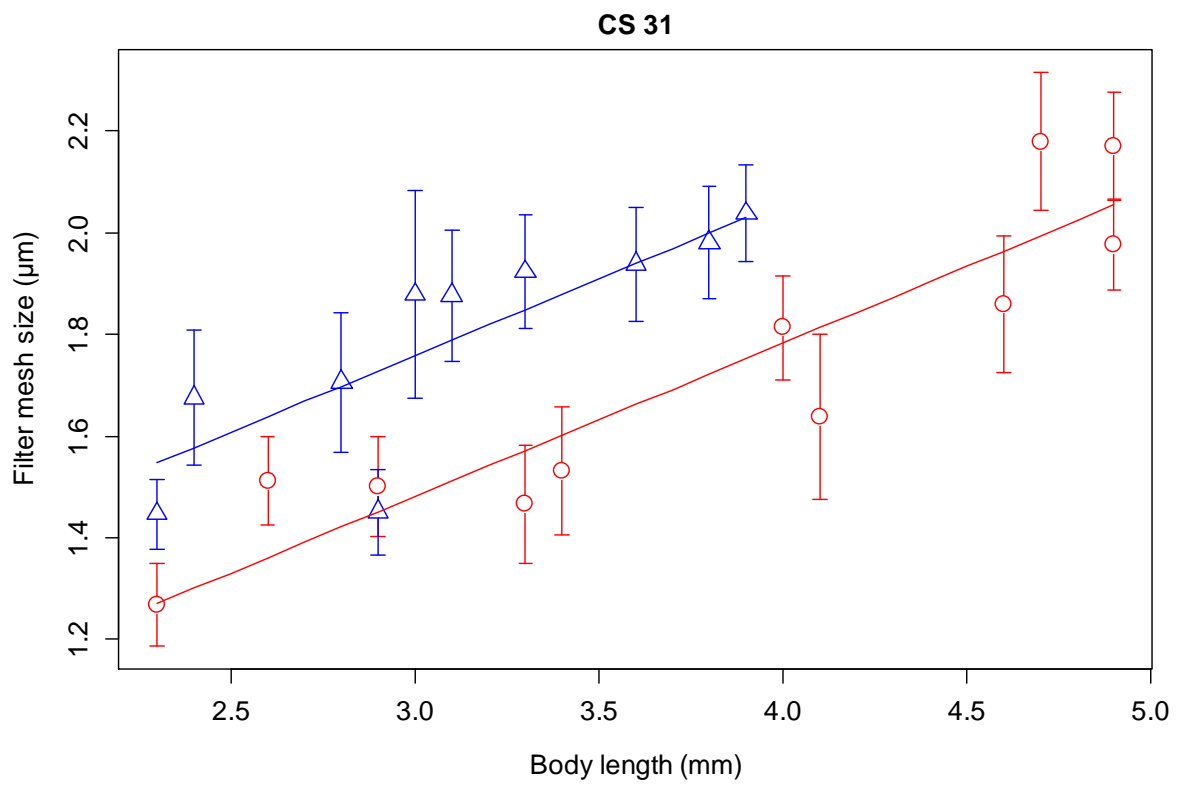

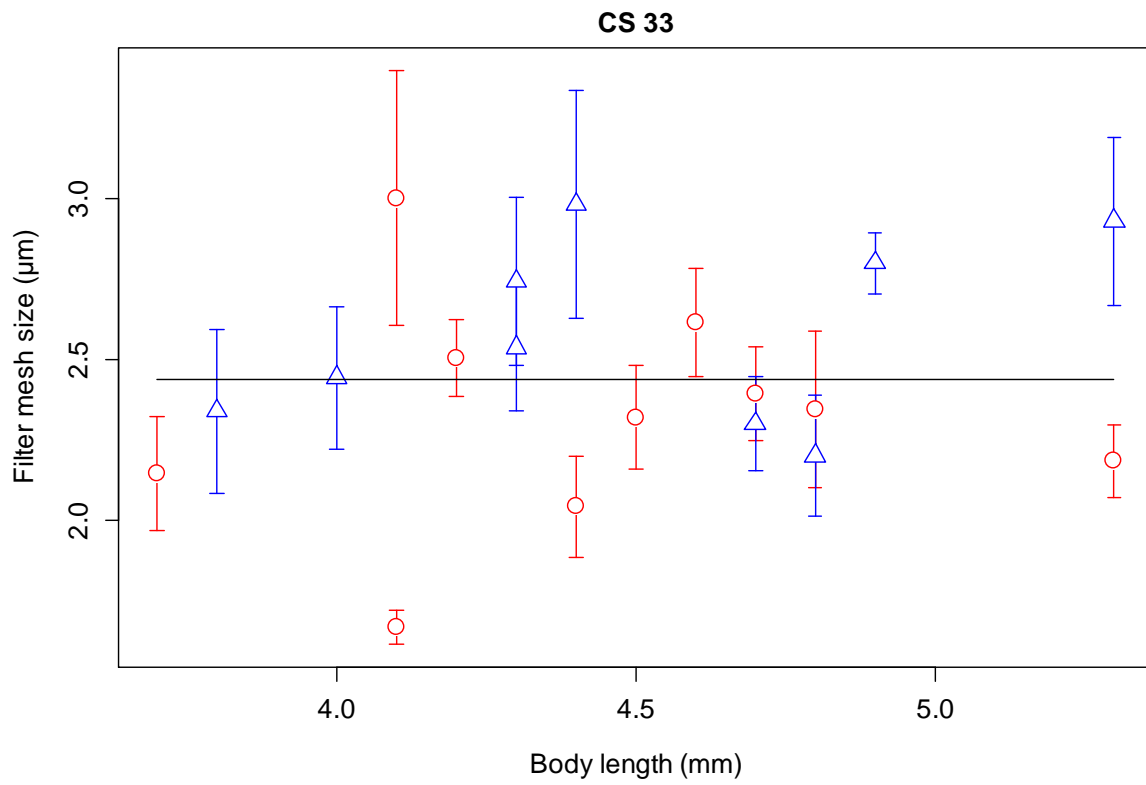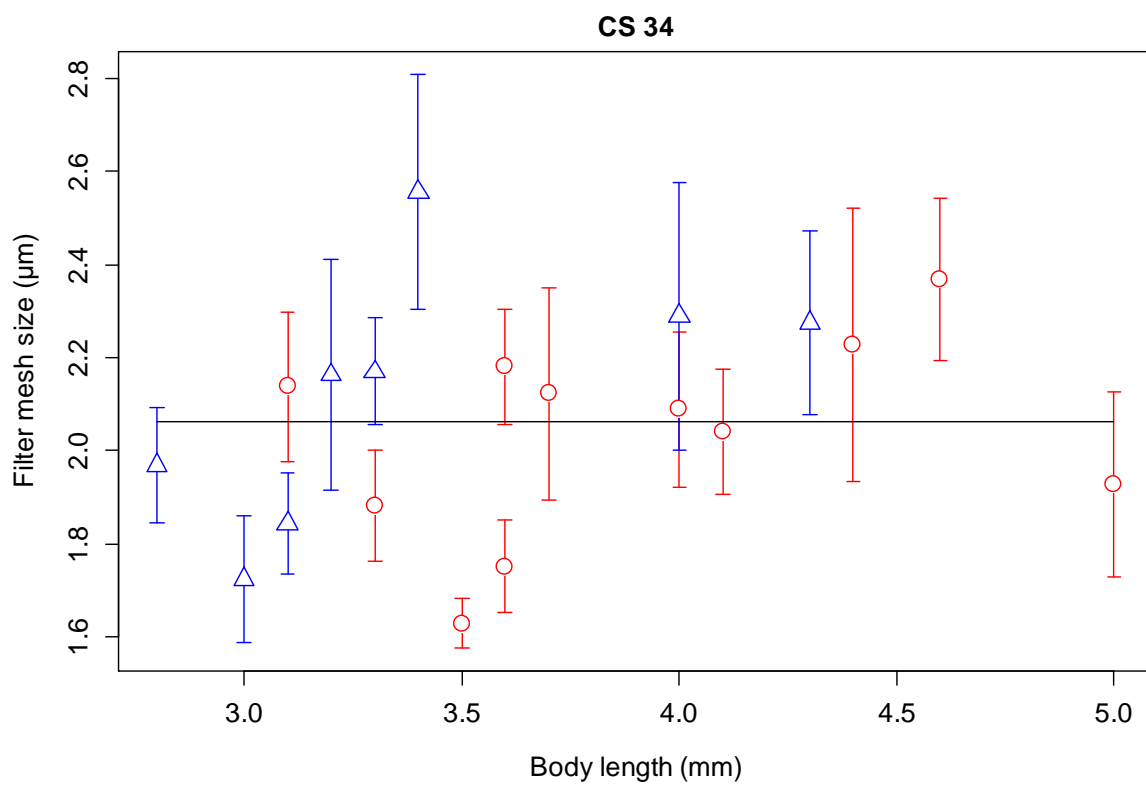

Supplement: Supplemental Information 2 — CC: C. curvispinum, CR: C. robustum, CS: C. sowinskyi. Red circles: females, blue triangles: males. The lines indicate the fixed effects of the optimal mixed-effect models (as defined in the text; Table S1-S2-S3). [file peerj-09-11245-s002.pdf]

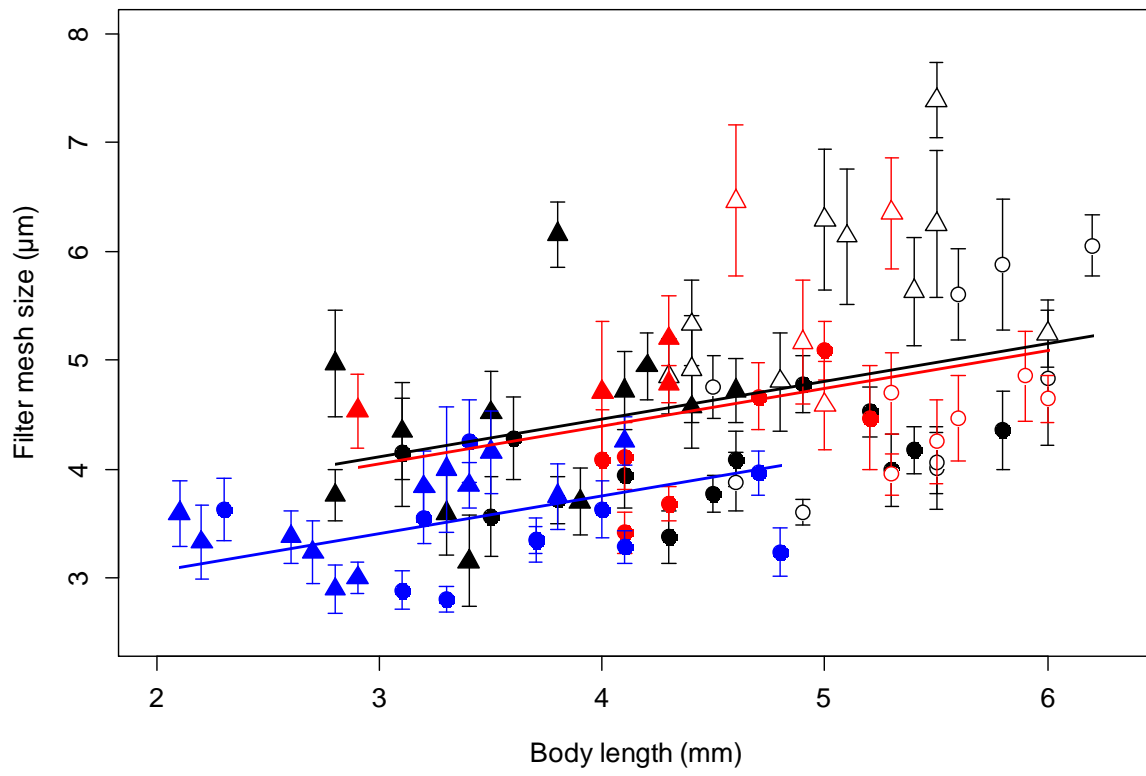

Supplement: Supplemental Information 3 — Colors indicate the years of collection; black: 2013 (empty symbols: sample no. 33, filled symbols: 34), red: 2014 (empty symbols: 35, filled symbols: 36), blue: 2017. Circles: females, triangles: males. The lines of corresponding colors represent the fixed effects (year and body length; the interaction was not significant) of the model including all five samples. The Tukey’s post-hoc test indicated significant differences between 2013- 2017 (estimate ± SE: -0.70 ± 0.22 μm, p = 0.0034), and 2014- 2017 (-0.64 ± 0.26 μm, p = 0.0317), but not between 2013-2014 (-0.06 ± 0.19 μm, p = 0.9473). Within-year differences (tested in separate models for 2013 and 2014) were explained by body length and sexes (2013: sex_male: 1.01 ± 0.22 μm, p < 0.001; body length: 0.63 ± 0.12 μm, p < 0.001; 2014: sex_male: 1.14 ± 0.24 μm, p = 0.0002; body length: 0.46 ± 0.16 μm, p = 0.0086). [file peerj-09-11245-s003.pdf]

Filter mesh size ( $\mu\text{m}$ )

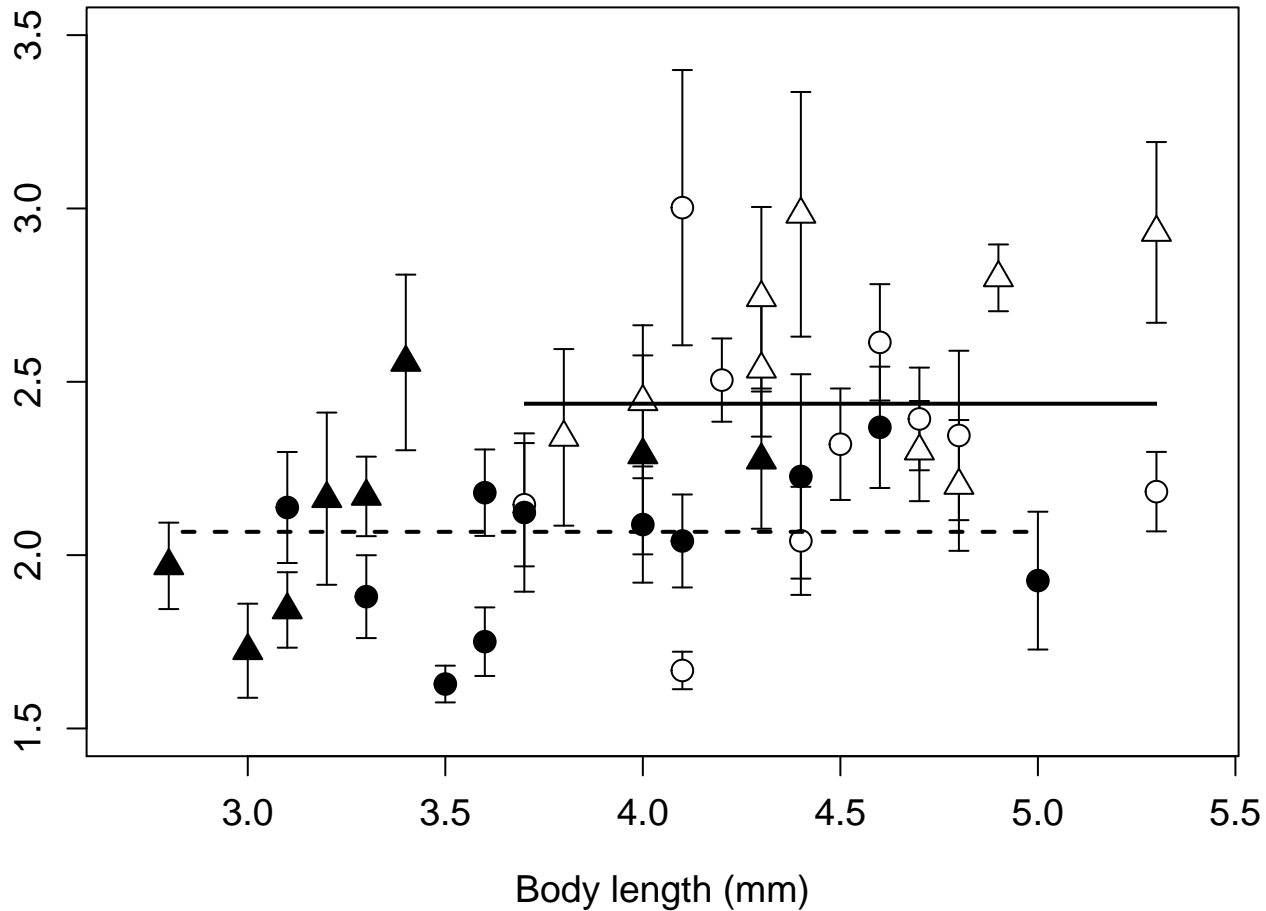

Supplement: Supplemental Information 4 — White: no. 33, 14.05.2013, black: no. 34, 31.07.2013. Circles: females, triangles: males. The lines represent the fixed effects of the optimal mixed-effect model including both samples. The difference between the samples was significant (mean ± SE: -0.37 ± 0.10, p = 0.0001). [file peerj-09-11245-s004.pdf]

Filter mesh size ( $\mu\text{m}$ )

6

5

4

3

2

3

4

5

Body length (mm)

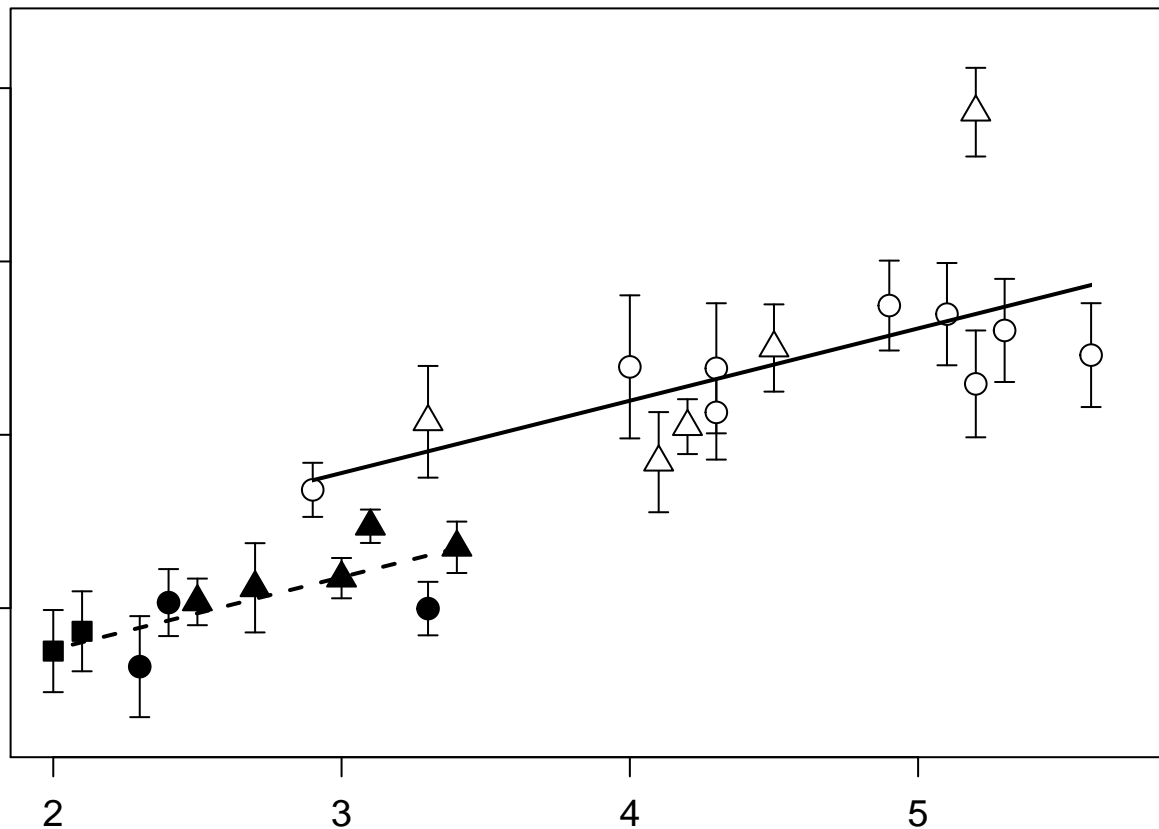

Supplement: Supplemental Information 5 — White: no. 38, 29.04.2016, black: no. 39, 04.11.2016. Circles: females, triangles: males, squares: juveniles. The lines represent the fixed effects of the optimal mixed-effect model including both samples. The body length effect (mean ± SE: 0.42 ± 0.10, p = 0.0001) and the difference between the samples (mean ± SE: -0.60 ± 0.22, p = 0.0138) were significant. [file peerj-09-11245-s005.pdf]
